# Antidepressants for the treatment of adults with major depressive disorder in the maintenance phase: a systematic review and network meta-analysis

**DOI:** 10.1038/s41380-022-01824-z

**Published:** 2022-10-17

**Authors:** Taro Kishi, Toshikazu Ikuta, Kenji Sakuma, Makoto Okuya, Masakazu Hatano, Yuki Matsuda, Nakao Iwata

**Affiliations:** 1 Department of Psychiatry, Fujita Health University School of Medicine, Toyoake, Aichi 470–1192 Japan; 2 Department of Communication Sciences and Disorders, School of Applied Sciences, University of Mississippi, University, Oxford, MS 38677 USA; 3 Department of Clinical Pharmacy, Fujita Health University School of Medicine, Toyoake, Aichi 470–1192 Japan; 4 Department of Psychiatry, The Jikei University School of Medicine, Minato-ku, Tokyo 105–8461 Japan

**Keywords:** Depression, Drug discovery

## Abstract

A systematic review and random-effects model network meta-analysis were conducted to compare the efficacy, acceptability, tolerability, and safety of antidepressants to treat adults with major depressive disorder (MDD) in the maintenance phase. This study searched the PubMed, Cochrane Library, and Embase databases and included only double-blind, randomized, placebo-controlled trials with an enrichment design: patients were stabilized on the antidepressant of interest during the open-label study and then randomized to receive the same antidepressant or placebo. The outcomes were the 6-month relapse rate (primary outcome, efficacy), all-cause discontinuation (acceptability), discontinuation due to adverse events (tolerability), and the incidence of individual adverse events. The risk ratio with a 95% credible interval was calculated. The meta-analysis comprised 34 studies (*n* = 9384, mean age = 43.80 years, and %females = 68.10%) on 20 antidepressants (agomelatine, amitriptyline, bupropion, citalopram, desvenlafaxine, duloxetine, escitalopram, fluoxetine, fluvoxamine, levomilnacipran, milnacipran, mirtazapine, nefazodone, paroxetine, reboxetine, sertraline, tianeptine, venlafaxine, vilazodone, and vortioxetine) and a placebo. In terms of the 6-month relapse rate, amitriptyline, citalopram, desvenlafaxine, duloxetine, fluoxetine, fluvoxamine, mirtazapine, nefazodone, paroxetine, reboxetine, sertraline, tianeptine, venlafaxine, and vortioxetine outperformed placebo. Compared to placebo, desvenlafaxine, paroxetine, sertraline, venlafaxine, and vortioxetine had lower all-cause discontinuation; however, sertraline had a higher discontinuation rate due to adverse events. Compared to placebo, venlafaxine was associated with a lower incidence of dizziness, while desvenlafaxine, sertraline, and vortioxetine were associated with a higher incidence of nausea/vomiting. In conclusion, desvenlafaxine, paroxetine, venlafaxine, and vortioxetine had reasonable efficacy, acceptability, and tolerability in the treatment of adults with stable MDD.

## Introduction

Major depressive disorder (MDD) is a common mental illness [1], with a 12-month prevalence of 4.4% worldwide [2]. Individuals with MDD in the acute phase undergo pharmacotherapy (e.g., antidepressant therapy) [3] or non-pharmacotherapy (e.g., psychotherapy [4] and electroconvulsive therapy) [5]. Relapse/recurrence rate of these patients is >85% within a decade of an index depressive episode and an average of ≥50% within 6 months of apparent clinical remission if the initially effective treatment is not continued [6]. Therefore, maintenance therapy is necessary to avoid relapse/recurrence [1].

Kato and colleagues recently conducted an important pairwise meta-analysis that included only double-blind, randomized placebo-controlled trials (DBRPCTs) with an enrichment design in which individuals with MDD were stabilized on the antidepressant of interest during the open-label study and then randomized to receive the same antidepressant or a placebo (40 studies, *n* = 8890) [7]. According to this meta-analysis, the antidepressant maintenance group had a significantly lower relapse rate than the antidepressant discontinuation group (odds ratio = 0.38, 95% confidence interval = 0.33–0.43, *p* < 0.00001). As the relapse rate remained unchanged in both the maintenance and discontinuation groups from 6 months to 1 year, Kato et al. concluded that antidepressant maintenance treatment for at least 6 months after remission is recommended to prevent relapse, with special attention to relapses and treatment failure during this 6-month period. Thanks to this excellent study, we conceived the new clinical question of which antidepressants were better in terms of efficacy, acceptability, tolerability, and safety for adult individuals with MDD as a maintenance treatment. A network meta-analysis on individuals with MDD in the acute phase demonstrated although some antidepressants (e.g., agomelatine, escitalopram, mirtazapine, paroxetine, and sertraline) have a relatively higher response rate and lower dropout rate than the others, fluvoxamine, reboxetine, and trazodone have been reported to have generally inferior efficacy and acceptability profiles compared with the other antidepressants [8]. This suggests that not all antidepressants have similar efficacies and acceptability in individuals with MDD in the acute phase. A network meta-analysis is a technique to compare three or more interventions simultaneously in a single analysis by combining both direct and indirect evidence across a network of studies [9]. A network meta-analysis also produces estimates of the relative effects between any pair of interventions in the network and usually yields more precise estimates than a single direct or indirect estimate, thereby allowing estimation of the ranking and hierarchy of interventions [9]. Results from a network meta-analysis cannot be obtained by a pairwise meta-analysis. Moreover, the previous pairwise meta-analyses for individuals with MDD in the maintenance phase did not evaluate the risk of individual adverse events of antidepressants [7, 10, 11]. To answer our clinical question, we conducted a systematic review and network meta-analysis on the 13 outcomes related to the efficacy, acceptability, tolerability, and safety of 20 antidepressants for the treatment of adults in the maintenance phase of MDD.

## Materials and methods

This study adhered to the Preferred Reporting Items for Systematic Reviews and Meta-Analyses (PRISMA) guidelines [12] (Table S1) and was registered on the Open Science Framework (https://osf.io/xwezp). At least two authors double-checked the accuracy of the literature search, data transfer, and calculations.

### Search strategy and inclusion criteria

A systematic literature review was conducted in accordance with the Population, Intervention, Comparison, Outcome strategy: the population comprised adults in the maintenance phase of MDD, the intervention was monotherapy with antidepressants, the comparator medication was a placebo, and the outcomes were described in the following section. The inclusion criteria were as follows: (1) DBRPCTs with a minimum duration of 12 weeks and (2) DBRPCTs with an enrichment design in which patients were stabilized on the antidepressant of interest during the open-label study and then randomized to receive the same antidepressant or a placebo. The following studies were excluded: (1) studies focusing on specific generations (e.g., children and/or adolescents or older individuals) because the efficacy and safety of antidepressants in children and older individuals differ from those in the general adult population [1]; (2) studies including individuals with a dual diagnosis of MDD and other disorders because these studies could lead to heterogeneity [1]; and (3) continuation studies in which individuals with acute symptoms were randomly assigned to treatment groups (i.e., the target population for a continuation study was individuals with MDD in the acute phase). In the present systematic review and meta-analysis, among adults with MDD who benefited symptomatically from antidepressant treatment (i.e., the target population for our systematic review and meta-analysis was individuals with MDD in the maintenance phase), the differences in relapse rates were compared between those who continued with the same antidepressant and those who discontinued the antidepressant. Information on the literature search is displayed in Fig. S1.

### Data synthesis, outcome measures, and data extraction

The primary outcome was the 6-month relapse rate (efficacy), and the secondary outcome was all-cause discontinuation (acceptability). Other outcomes included discontinuation due to adverse events (tolerability) and the incidence of individual adverse events (safety). If at least five studies have data sufficient to perform a network meta-analysis for a specific safety outcome, a network meta-analysis was conducted for the safety outcome. In the International Classification of Diseases 11th Revision [13], recurrent depressive disorder is defined by a history of at least two depressive episodes with an interval of several months without substantial mood disturbance. In the present study, the term “relapse” is used for convenience rather than “recurrence” similar to the previous study [7], because few studies in this meta-analysis included cases in which worsening of symptoms during the study period was considered a recurrence. The definitions of relapse for each included study are presented in Table S2, and the data synthesis results are shown in Table S3. To avoid unit-of-analysis errors in studies involving two or more treatment arms of the same drug at different doses, data from the treatment arms were pooled for analysis [9]. The extracted data were analyzed based on intention-to-treat or modified intention-to-treat principles. If necessary data were missing from the studies, we searched for them in published systematic review articles; we also attempted to contact the original investigators in order to obtain previously unpublished data.

### Meta-analysis methods

Both pairwise [14] and Bayesian network meta-analyses [15] were performed using the random-effects model [16]. Because all of the outcomes in our study were dichotomous, risk ratios (RRs) with 95% credible intervals (CrIs) were calculated as effect sizes. Network heterogeneity was assessed using *τ*² statistics. In pairwise meta-analyses, heterogeneity was assessed using *I*^2^ statistics. A statistical evaluation of incoherence was not possible because there was no head-to-head study comparing different antidepressants. The treatments for each outcome were ranked using the surface under the curve cumulative ranking probabilities. The methodological quality of the included studies was evaluated using the Cochrane risk of bias tool for randomized trials (ROB2) (https://www.riskofbias.info/welcome/rob-2-0-tool). The assumption of transitivity was tested by extracting potential effect modifiers such as sample size, duration of study, and mean age and comparing their distribution across comparisons in the network. We determined whether the distribution differences were large enough to threaten the validity of the analysis by comparing the distribution of these possible effect modifiers across treatments included in the network meta-analysis using the Kruskal–Wallis test (continuous variables), the Pearson chi-squared test or the Fisher exact test (categorical variables) and by assessing their actual impact on the treatment effect through meta-regression analyses [17, 18]. A meta-regression analysis was performed to determine the relationship of potentially confounding factors (e.g., mean age, proportion of females, number of episodes, total number of participants, patient status, publication year, sponsorship, duration of preliminary phase, country, discontinuation methods, risk of bias, antidepressant class, dosage schedule, and antidepressant dose) to the magnitude of the effect on the primary outcome. Funnel plots were created to investigate potential publication bias. Finally, to assess the credibility of the findings of each network meta-analysis, the findings were incorporated into the Confidence in Network Meta-Analysis (CINeMA) application, which is an adaptation of the Grading of Recommendations Assessment, Development, and Evaluation approach [19–21].

## Results

### Study characteristics

The literature search and selection strategy are depicted in Fig. S1. The initial search retrieved 148 articles, 50 of which were excluded as duplicates, 95 were excluded based on a review of the abstract and/or title, and three were included in our study [22–24]. In addition, 31 studies were retrieved [25–55] by manually searching the reference lists of previous review article [7]. There were no additional studies found in the clinical trial registers. Finally, the present review included a total of 34 DBRPCTs comprising 9384 patients with MDD (mean age = 43.80 years and %females = 68.10%). The characteristics of the 34 DBRPCTs included are summarized in Table S4. The average length of the study was 40.94 ± 16.27 weeks. Adults in the maintenance group were administered agomelatine (*K* = 2), amitriptyline (*K* = 1), bupropion (*K* = 1), citalopram (*K* = 3), desvenlafaxine (*K* = 2), duloxetine (*K* = 2), escitalopram (*K* = 1), fluoxetine (*K* = 4), fluvoxamine (*K* = 1), levomilnacipran (*K* = 2), milnacipran (*K* = 1), mirtazapine (*K* = 1), nefazodone (*K* = 1), paroxetine (*K* = 2), reboxetine (*K* = 1), sertraline (*K* = 2), tianeptine (*K* = 1), venlafaxine (*K* = 3), vilazodone (*K* = 1), and vortioxetine (*K* = 2). In 32 studies, participants in the acute study were required to have a scale-derived minimum of symptoms at baseline. However, one study lacked such a criterion, while another lacked detailed information on the criterion. Although 20 of the studies included only outpatients, six included both inpatients and outpatients, and the remaining eight did not report the status. All studies employed operationalized criteria such as those found in Diagnostic and Statistical Manual of Mental Disorders [56]. For the placebo group, the drug was discontinued abruptly (7 studies) and gradually (12 studies), and the remaining 15 studies did not report the detailed method of drug discontinuation. In addition, 31 studies were sponsored by the industry. The distribution of potential effect modifiers was similar across the comparisons in the network (Table S5). In at least one domain of the ROB2 tool, no studies were determined to be at high risk of bias (Table S6).

### Network meta-analysis results

The network meta-analysis results are shown in Appendices S1–S13.

### Efficacy

**Fig. 1:**
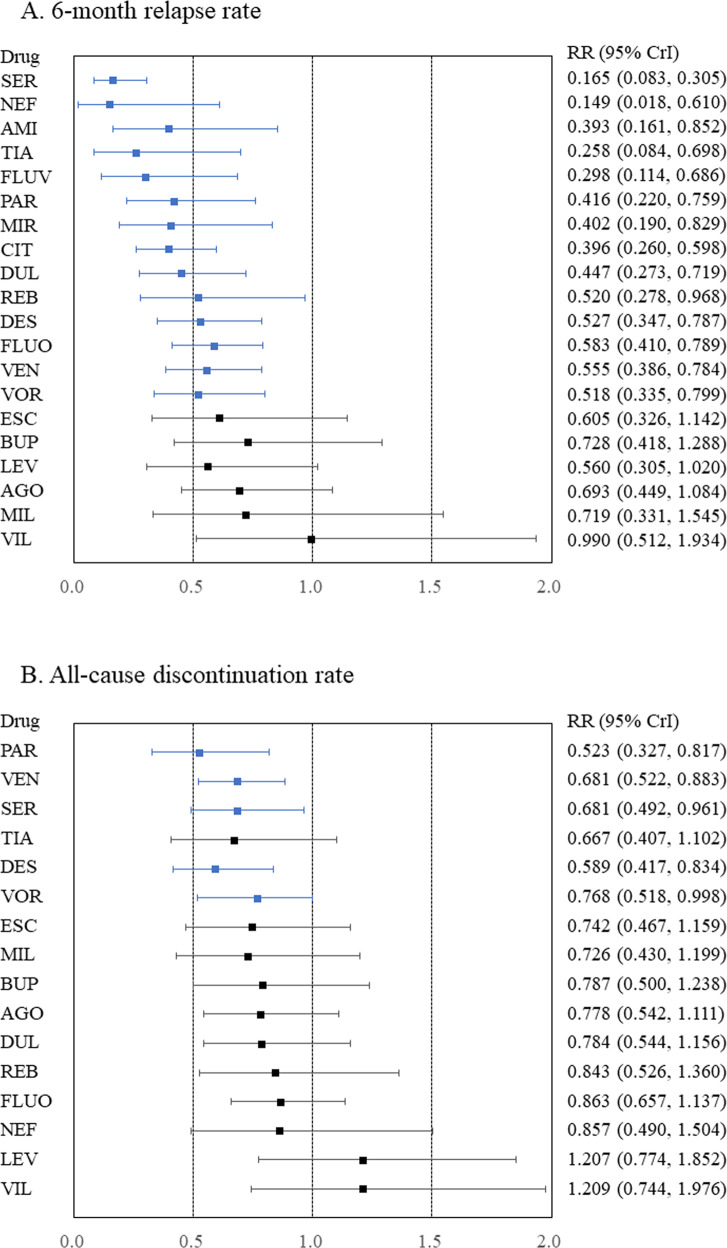
Forest plots for the 6-month relapse and all-cause discontinuation rates. **A** 6-month relapse rate and **B** all-cause discontinuation rate. Medications were compared to a placebo. Colors indicate the presence or absence of a statistically significant difference, with blue indicating that the drug was superior to the placebo and black indicating that the drug was comparable to the placebo. 95% CrI 95% credible interval, AGO agomelatine, AMI amitriptyline, BUP bupropion, CIT citalopram, DES desvenlafaxine, DUL duloxetine, ESC escitalopram, FLUO fluoxetine, FLUV fluvoxamine, LEV levomilnacipran, MIL milnacipran, MIR mirtazapine, NEF nefazodone, PAR paroxetine, REB reboxetine, RR risk ratio, SER sertraline, TIA tianeptine, VEN venlafaxine, VIL vilazodone, VOR vortioxetine.

In terms of the 6-month relapse rate, amitriptyline, citalopram, desvenlafaxine, duloxetine, fluoxetine, fluvoxamine, mirtazapine, nefazodone, paroxetine, reboxetine, sertraline, tianeptine, venlafaxine, and vortioxetine outperformed the placebo (Fig. 1, Appendix S1), with RRs (95% CrIs) ranging from 0.149 (0.018–0.610) for nefazodone to 0.583 (0.410–0.789) for fluoxetine. In addition, citalopram, fluvoxamine, and tianeptine outperformed vilazodone. Moreover, nefazodone outperformed agomelatine, bupropion, and vilazodone. Furthermore, sertraline outperformed agomelatine, bupropion, citalopram, desvenlafaxine, duloxetine, escitalopram, fluoxetine, levomilnacipran, milnacipran, paroxetine, reboxetine, venlafaxine, vilazodone, and vortioxetine. Global heterogeneity was moderate. A funnel plot for this outcome, although no comparisons included at least 10 studies, is displayed in Appendix S1. On meta-regression analyses, no potentially confounding factors were associated with the RR of the primary outcome (Appendix S1). Heterogeneity was not strongly reduced despite adjustments for any potentially confounding factors in a meta-regression (Appendix S1). Thus, no clear evidence of violations of the transitivity assumption for any of the potential effect modifiers analyzed was found (Table S5 and Appendix S1).

### Acceptability

Compared to placebo, desvenlafaxine, paroxetine, sertraline, venlafaxine, and vortioxetine had lower all-cause discontinuation (Fig. 1, Appendix S2), with RRs (95% CrIs) ranging from 0.523 (0.327–0.817) for paroxetine to 0.768 (0.518–0.998) for vortioxetine. Desvenlafaxine, paroxetine, and venlafaxine outperformed levomilnacipran and vilazodone. Sertraline also outperformed levomilnacipran. Global heterogeneity was moderate.

### Tolerability and safety outcomes

**Fig. 2:**
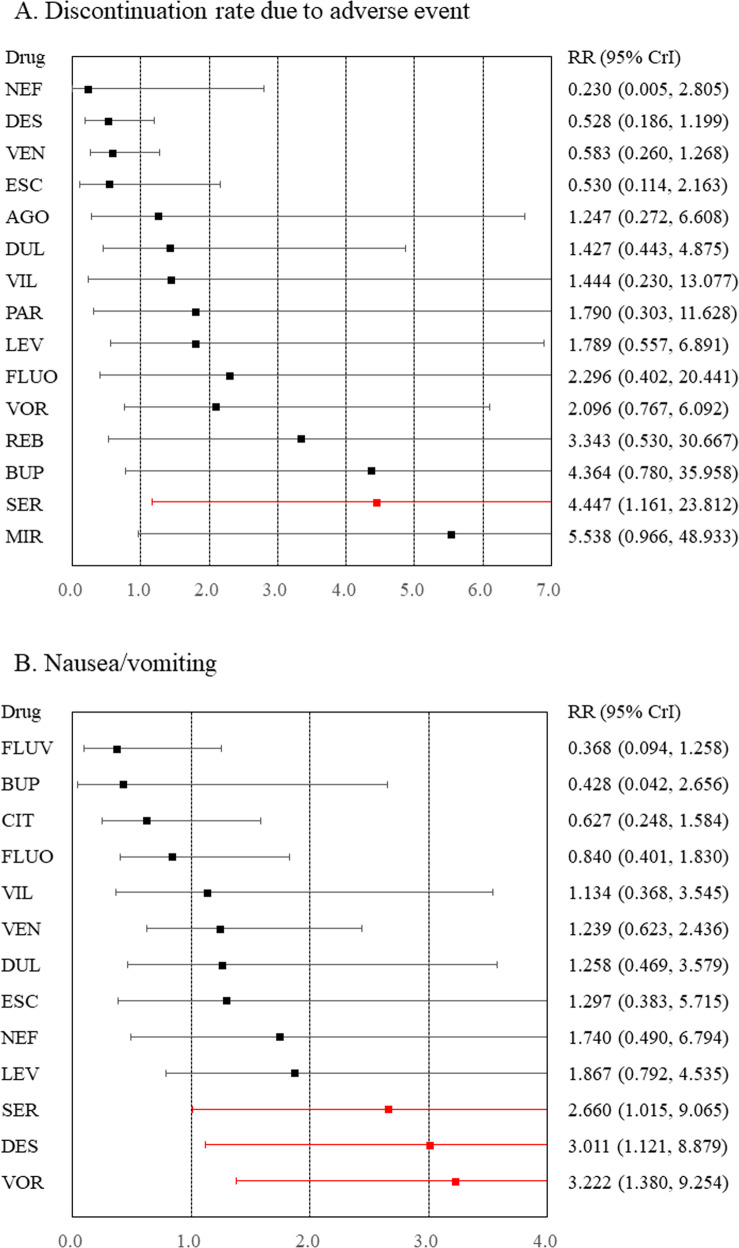
Forest plots for discontinuation rate due to adverse events and nausea/vomiting. **A** Discontinuation rate due to adverse events and **B** nausea/vomiting. Medications were compared with a placebo. Colors indicate the presence or absence of a statistically significant difference, with red indicating that the drug was inferior to the placebo and black indicating that the drug was comparable to the placebo. 95% CrI 95% credible interval, AGO agomelatine, BUP bupropion, CIT citalopram, DES desvenlafaxine, DUL duloxetine, ESC escitalopram, FLUO fluoxetine, FLUV fluvoxamine, LEV levomilnacipran, MIR mirtazapine, NEF nefazodone, PAR paroxetine, REB reboxetine, RR risk ratio, SER sertraline, VEN venlafaxine, VIL vilazodone, VOR vortioxetine.

Compared to placebo, sertraline was associated with a higher rate of discontinuation due to adverse events (Fig. 2 and Appendix S3). Compared to placebo, although desvenlafaxine, sertraline, and vortioxetine were associated with a higher incidence of nausea/vomiting (Fig. 2 and Appendix S4), venlafaxine was associated with a lower incidence of dizziness (Appendix S5). Compared to placebo, any antidepressants were not associated with an increased incidence of headache, somnolence, insomnia, dry mouth, constipation, sweating, weight gain, or sexual dysfunction (Appendices S6–13).

### Heterogeneity, inconsistency, and network meta-analysis results graded using the CINeMA application

Global heterogeneity was rated as moderate for all outcomes, except for constipation and sexual dysfunction, for which global heterogeneity was rated as high (Appendices S1–13). A considerable local heterogeneity was observed for the majority of outcomes in specific comparisons. Statistical evaluation of incoherence was impossible due to the absence of a head-to-head study comparing various antidepressants. Between network meta-analysis and pairwise meta-analysis, results showed differences in the following in comparison to placebo: agomelatine and levomilnacipran for the 6-month relapse rates, tianeptine for all-cause discontinuation rate, desvenlafaxine and mirtazapine for discontinuation rates due to adverse events, sertraline for nausea/vomiting, desvenlafaxine for dizziness, duloxetine for dry mouth, citalopram for constipation, and sertraline for sexual dysfunction. The within-study bias was rated as “some concerns” for all comparisons. Because funnel plots with fewer than 10 studies were not meaningful [9], all comparisons for publication bias were rated as “suspected,” and any inconsistency could not be evaluated. Furthermore, the comparison was downgraded one level if it was based only on indirect evidence. Therefore, the confidence in the evidence for all comparisons other than vortioxetine versus placebo (low) in terms of the primary outcome was rated as “very low (Appendix S1).”

## Discussion

To the best of our knowledge, this is the first systematic review and network meta-analysis to investigate which antidepressant has the best balance of efficacy and acceptability for the treatment of adult individuals with MDD in the maintenance phase. Although desvenlafaxine, paroxetine, sertraline, venlafaxine, and vortioxetine had the best balance, sertraline was not well tolerated due to its association with nausea/vomiting. Therefore, desvenlafaxine, paroxetine, venlafaxine, and vortioxetine may be beneficial to individuals with MDD in the maintenance phase. However, desvenlafaxine and vortioxetine were associated with a risk of nausea/vomiting in adults with MDD in the maintenance phase as well as in the acute phase [57]. The efficacy, acceptability, tolerability, and safety of the treatment of MDD in the maintenance phase should be carefully considered as treatments prescribed for an acute depressive episode are typically continued into maintenance. Results of a network meta-analysis of adults with acute MDD also revealed that desvenlafaxine, paroxetine, venlafaxine, and vortioxetine had good efficacy and acceptability [8].

In contrast, the findings of the present network meta-analysis suggest that agomelatine, bupropion, escitalopram, levomilnacipran, milnacipran, and vilazodone did not outperform the placebo in terms of 6-month relapse rate. The original DBRPCTs reported that although vilazodone did not differ from placebo in terms of relapse rate at the study-endpoint [23], escitalopram and bupropion were superior to placebo [43, 55]. Two DBRPCTs on agomelatine had inconsistent results [32, 33]. One DBRPCT reported that levomilnacipran outperformed placebo in terms of relapse rate at the study-endpoint [22], while another DBRPCT did not report the statistical result of the outcome [49]; one trial investigating milnacipran also did not report the statistical results [47]. Our pairwise meta-analysis showed that agomelatine and levomilnacipran outperformed the placebo (Appendix S1). Due to the small number of individuals in these antidepressant trials, the 95% CrIs for the primary outcome in the network meta-analysis might be wider. As a result, our network meta-analysis might not be able to detect the significant differences between these antidepressants and placebo.

A previous meta-regression analysis based on a pairwise meta-analysis showed that the effect size of the relapse rates was greater for tricyclics, selective serotonin reuptake inhibitors, and other newer agents, in that order, compared with the placebo [7]. However, our study did not demonstrate this trend (Appendix S1). Through a network meta-analysis, the relative effects can be estimated using any pair of interventions in the network simultaneously as well as the ranking and hierarchy of the interventions based on effectiveness [9, 58]. Thus, when comparing the efficacy of individual antidepressants, a network meta-analysis is likely to yield more robust results than a pairwise meta-analysis.

There are some limitations to this study. First, the number of participants and DBRPCTs for some antidepressants, especially for tricyclic antidepressants, is small. The results of the present meta-analysis for some antidepressants were based on only one study. Second, important clinical issues regarding treatment decision-making in routine clinical practice (e.g., monotherapy or combination of antidepressants with nonpharmacological treatments) were not covered. A Finnish nationwide cohort study of individuals with severe MDD requiring hospitalization (mean follow-up time, 7.9 ± 5.3 years) found that lithium treatment was associated with the lowest risk of hospital readmission in patients with severe unipolar depression compared with other pharmacological treatments such as antidepressant and antipsychotics [59, 60]. Sim and colleagues also reported that psychotherapy may have long-term benefits, particularly for patients with at least three previous major depressive episodes [10]. However, because there were no DBRPCT with an enrichment design for those treatments, our study did not evaluate these treatments for individuals with MDD. Third, due to a lack of available data, our study did not include some important antidepressant side effects such as agitation.

In conclusion, antidepressants such as desvenlafaxine, paroxetine, venlafaxine, and vortioxetine had balanced efficacy, acceptability, and tolerability in the treatment of adults with MDD in the maintenance phase. However, desvenlafaxine and vortioxetine had a risk of nausea/vomiting in adults with MDD in both the maintenance and acute phases.

## Supplementary information

Supplementary materials

## Data Availability

The current study data were reported in articles cited in this paper.

## References

[1] Herrman H, Patel V, Kieling C, Berk M, Buchweitz C, Cuijpers P (2022). Time for united action on depression: a Lancet-World Psychiatric Association Commission. Lancet.

[2] WHO. Depression and other common mental disorders: global health estimates. World Health Organization 2017; Geneva.

[3] Kennedy SH, Lam RW, McIntyre RS, Tourjman SV, Bhat V, Blier P (2016). Canadian Network for Mood and Anxiety Treatments (CANMAT) 2016 clinical guidelines for the management of adults with major depressive disorder: section 3. Pharmacological treatments. Can J Psychiatry.

[4] Parikh SV, Quilty LC, Ravitz P, Rosenbluth M, Pavlova B, Grigoriadis S (2016). Canadian Network for Mood and Anxiety Treatments (CANMAT) 2016 clinical guidelines for the management of adults with major depressive disorder: section 2. Psychological treatments. Can J Psychiatry.

[5] Milev RV, Giacobbe P, Kennedy SH, Blumberger DM, Daskalakis ZJ, Downar J (2016). Canadian Network for Mood and Anxiety Treatments (CANMAT) 2016 clinical guidelines for the management of adults with major depressive disorder: section 4. Neurostimulation treatments. Can J Psychiatry.

[6] Baldessarin IR Chemotherapy in Psychiatry, 3rd edition. Springer Press 2013; New York.

[7] Kato M, Hori H, Inoue T, Iga J, Iwata M, Inagaki T (2021). Discontinuation of antidepressants after remission with antidepressant medication in major depressive disorder: a systematic review and meta-analysis. Mol Psychiatry.

[8] Cipriani A, Furukawa TA, Salanti G, Chaimani A, Atkinson LZ, Ogawa Y (2018). Comparative efficacy and acceptability of 21 antidepressant drugs for the acute treatment of adults with major depressive disorder: a systematic review and network meta-analysis. Lancet.

[9] Higgins J, Thomas J, Chandler J, Cumpston M, Li T, Page M, et al. Cochrane Handbook for Systematic Reviews of Interventions version 6.2. wwwtrainingcochraneorg/handbook 2021.

[10] Sim K, Lau WK, Sim J, Sum MY, Baldessarini RJ. Prevention of relapse and recurrence in adults with major depressive disorder: systematic review and meta-analyses of controlled trials. Int J Neuropsychopharmacol 2015;19:pyv076. 10.1093/ijnp/pyv076 PMC4772815 26152228

[11] Glue P, Donovan MR, Kolluri S, Emir B (2010). Meta-analysis of relapse prevention antidepressant trials in depressive disorders. Aust N. Z J Psychiatry.

[12] Hutton B, Salanti G, Caldwell DM, Chaimani A, Schmid CH, Cameron C (2015). The PRISMA extension statement for reporting of systematic reviews incorporating network meta-analyses of health care interventions: checklist and explanations. Ann Intern Med.

[13] WHO. International Statistical Classification of Diseases and Related Health Problems 11th. World Health Organization.

[14] Rücker G, Schwarzer G, Krahn U, König J netmeta: Network Meta-Analysis using Frequentist Methods (R package version 0.9-5). https://CRANR-projectorg/package=netmeta 2017; (accessed March 14, 2020).

[15] van Valkenhoef G, Lu G, de Brock B, Hillege H, Ades AE, Welton NJ (2012). Automating network meta-analysis. Res Synth Methods.

[16] DerSimonian R, Laird N (1986). Meta-analysis in clinical trials. Controlled Clin trials.

[17] Cipriani A, Higgins JP, Geddes JR, Salanti G (2013). Conceptual and technical challenges in network meta-analysis. Ann Intern Med.

[18] Ostuzzi G, Bertolini F, Tedeschi F, Vita G, Brambilla P, Del Fabro L (2022). Oral and long-acting antipsychotics for relapse prevention in schizophrenia-spectrum disorders: a network meta-analysis of 92 randomized trials including 22,645 participants. World Psychiatry.

[19] Salanti G, Del Giovane C, Chaimani A, Caldwell DM, Higgins JP (2014). Evaluating the quality of evidence from a network meta-analysis. PLoS One.

[20] Nikolakopoulou A, Higgins JPT, Papakonstantinou T, Chaimani A, Del Giovane C, Egger M (2020). CINeMA: An approach for assessing confidence in the results of a network meta-analysis. PLoS Med.

[21] Papakonstantinou T, Nikolakopoulou A, Higgins JPT, Egger M, Salanti G (2020). CINeMA: Software for semiautomated assessment of the confidence in the results of network meta-analysis. Campbell Syst Rev.

[22] Durgam S, Chen C, Migliore R, Prakash C, Thase ME (2019). Relapse prevention with levomilnacipran ER in adults with major depressive disorder: A multicenter, randomized, double-blind, placebo-controlled study. Depress Anxiety.

[23] Durgam S, Gommoll C, Migliore R, Chen C, Chang CT, Aguirre M (2018). Relapse prevention in adults with major depressive disorder treated with vilazodone: a randomized, double-blind, placebo-controlled trial. Int Clin Psychopharmacol.

[24] Thase ME, Jacobsen PL, Hanson E, Xu R, Tolkoff M, Murthy NV (2022). Vortioxetine 5, 10, and 20 mg significantly reduces the risk of relapse compared with placebo in patients with remitted major depressive disorder: The RESET study. J Affect Disord.

[25] Boulenger JP, Loft H, Florea I (2012). A randomized clinical study of Lu AA21004 in the prevention of relapse in patients with major depressive disorder. J Psychopharmacol.

[26] Dalery J, Dagens-Lafont V, De, Bodinat C (2001). Efficacy of tianeptine vs placebo in the long-term treatment (16.5 months) of unipolar major recurrent depression*. Hum Psychopharmacol.

[27] Dekker J, Jonghe F, Tuynman H (2000). The use of anti-depressants after recovery from depression. Eur J Psychiatry.

[28] Dobson KS, Hollon SD, Dimidjian S, Schmaling KB, Kohlenberg RJ, Gallop RJ (2008). Randomized trial of behavioral activation, cognitive therapy, and antidepressant medication in the prevention of relapse and recurrence in major depression. J Consult Clin Psychol.

[29] Doogan DP, Caillard V (1992). Sertraline in the prevention of depression. Br J Psychiatry.

[30] Feiger AD, Bielski RJ, Bremner J, Heiser JF, Trivedi M, Wilcox CS (1999). Double-blind, placebo-substitution study of nefazodone in the prevention of relapse during continuation treatment of outpatients with major depression. Int Clin Psychopharmacol.

[31] Gilaberte I, Montejo AL, de la Gandara J, Perez-Sola V, Bernardo M, Massana J (2001). Fluoxetine in the prevention of depressive recurrences: a double-blind study. J Clin Psychopharmacol.

[32] Goodwin GM, Boyer P, Emsley R, Rouillon F, de Bodinat C (2013). Is it time to shift to better characterization of patients in trials assessing novel antidepressants? An example of two relapse prevention studies with agomelatine. Int Clin Psychopharmacol.

[33] Goodwin GM, Emsley R, Rembry S, Rouillon F (2009). Agomelatine Study G. Agomelatine prevents relapse in patients with major depressive disorder without evidence of a discontinuation syndrome: a 24-week randomized, double-blind, placebo-controlled trial. J Clin Psychiatry.

[34] Hochstrasser B, Isaksen PM, Koponen H, Lauritzen L, Mahnert FA, Rouillon F (2001). Prophylactic effect of citalopram in unipolar, recurrent depression: placebo-controlled study of maintenance therapy. Br J Psychiatry.

[35] Keller MB, Kocsis JH, Thase ME, Gelenberg AJ, Rush AJ, Koran L (1998). Maintenance phase efficacy of sertraline for chronic depression: a randomized controlled trial. JAMA.

[36] Kocsis JH, Thase ME, Trivedi MH, Shelton RC, Kornstein SG, Nemeroff CB (2007). Prevention of recurrent episodes of depression with venlafaxine ER in a 1-year maintenance phase from the PREVENT Study. J Clin Psychiatry.

[37] McGrath PJ, Stewart JW, Quitkin FM, Chen Y, Alpert JE, Nierenberg AA (2006). Predictors of relapse in a prospective study of fluoxetine treatment of major depression. Am J Psychiatry.

[38] Montgomery SA, Dunbar G (1993). Paroxetine is better than placebo in relapse prevention and the prophylaxis of recurrent depression. Int Clin Psychopharmacol.

[39] Montgomery SA, Entsuah R, Hackett D, Kunz NR, Rudolph RL (2004). Venlafaxine 335 Study G. Venlafaxine versus placebo in the preventive treatment of recurrent major depression. J Clin Psychiatry.

[40] Montgomery SA, Rasmussen JG, Tanghoj P (1993). A 24-week study of 20 mg citalopram, 40 mg citalopram, and placebo in the prevention of relapse of major depression. Int Clin Psychopharmacol.

[41] Perahia DG, Gilaberte I, Wang F, Wiltse CG, Huckins SA, Clemens JW (2006). Duloxetine in the prevention of relapse of major depressive disorder: double-blind placebo-controlled study. Br J Psychiatry.

[42] Perahia DG, Maina G, Thase ME, Spann ME, Wang F, Walker DJ (2009). Duloxetine in the prevention of depressive recurrences: a randomized, double-blind, placebo-controlled trial. J Clin Psychiatry.

[43] Rapaport MH, Bose A, Zheng H (2004). Escitalopram continuation treatment prevents relapse of depressive episodes. J Clin Psychiatry.

[44] Rickels K, Montgomery SA, Tourian KA, Guelfi JD, Pitrosky B, Padmanabhan SK (2010). Desvenlafaxine for the prevention of relapse in major depressive disorder: results of a randomized trial. J Clin Psychopharmacol.

[45] Robert P, Montgomery SA (1995). Citalopram in doses of 20-60 mg is effective in depression relapse prevention: a placebo-controlled 6 month study. Int Clin Psychopharmacol.

[46] Rosenthal JZ, Boyer P, Vialet C, Hwang E, Tourian KA (2013). Efficacy and safety of desvenlafaxine 50 mg/d for prevention of relapse in major depressive disorder:a randomized controlled trial. J Clin Psychiatry.

[47] Rouillon F, Warner B, Pezous N, Bisserbe JC (2000). Milnacipran efficacy in the prevention of recurrent depression: a 12-month placebo-controlled study. Milnacipran recurrence prevention study group. Int Clin Psychopharmacol.

[48] Schmidt ME, Fava M, Robinson JM, Judge R (2000). The efficacy and safety of a new enteric-coated formulation of fluoxetine given once weekly during the continuation treatment of major depressive disorder. J Clin Psychiatry.

[49] Shiovitz T, Greenberg WM, Chen C, Forero G, Gommoll CP (2014). A randomized, double-blind, placebo-controlled trial of the efficacy and safety of levomilnacipran ER 40-120mg/day for prevention of relapse in patients with major depressive disorder. Innov Clin Neurosci.

[50] Simon JS, Aguiar LM, Kunz NR, Lei D (2004). Extended-release venlafaxine in relapse prevention for patients with major depressive disorder. J Psychiatr Res.

[51] Stein MK, Rickels K, Weise CC (1980). Maintenance therapy with amitriptyline: a controlled trial. Am J Psychiatry.

[52] Terra JL, Montgomery SA (1998). Fluvoxamine prevents recurrence of depression: results of a long-term, double-blind, placebo-controlled study. Int Clin Psychopharmacol.

[53] Thase ME, Nierenberg AA, Keller MB, Panagides J (2001). Relapse Prevention Study G. Efficacy of mirtazapine for prevention of depressive relapse: a placebo-controlled double-blind trial of recently remitted high-risk patients. J Clin Psychiatry.

[54] Versiani M, Mehilane L, Gaszner P, Arnaud-Castiglioni R (1999). Reboxetine, a unique selective NRI, prevents relapse and recurrence in long-term treatment of major depressive disorder. J Clin Psychiatry.

[55] Weihs KL, Houser TL, Batey SR, Ascher JA, Bolden-Watson C, Donahue RM (2002). Continuation phase treatment with bupropion SR effectively decreases the risk for relapse of depression. Biol Psychiatry.

[56] APA. Diagnostic and Statistical Manual of Mental Disorders. American Psychiatric Association; Washington, D. C.

[57] Oliva V, Lippi M, Paci R, Del Fabro L, Delvecchio G, Brambilla P (2021). Gastrointestinal side effects associated with antidepressant treatments in patients with major depressive disorder: A systematic review and meta-analysis. Prog Neuropsychopharmacol Biol Psychiatry.

[58] Leucht S, Chaimani A, Cipriani AS, Davis JM, Furukawa TA, Salanti G (2016). Network meta-analyses should be the highest level of evidence in treatment guidelines. Eur Arch Psychiatry Clin Neurosci.

[59] Tiihonen J (2017). Use of lithium in patients with unipolar depression - Author’s reply. Lancet Psychiatry.

[60] Tiihonen J, Tanskanen A, Hoti F, Vattulainen P, Taipale H, Mehtala J (2017). Pharmacological treatments and risk of readmission to hospital for unipolar depression in Finland: a nationwide cohort study. Lancet Psychiatry.

