# Supplementary material for: Antidepressants for the treatment of adults with major depressive disorder in the maintenance phase: a systematic review and network meta-analysis
Source: Mol Psychiatry. 2022 Oct 17;28(1):402–9. doi: 10.1038/s41380-022-01824-z (PMC9812779; doi:10.1038/s41380-022-01824-z)

Figure S1. Flow diagram of literature search.

We searched PubMed, Cochrane Library, and Embase databases for studies published before May 22, 2022. The search terms for PubMed and Cochrane Library included (major depressi*) AND (random*) AND (double-blind) AND (reccuren* OR relapse) AND (placebo). No language restriction was applied to the literature search. The search terms for Embase included ('major depression'/exp OR 'major depression') AND ('randomized controlled trial'/exp OR 'randomized controlled trial') AND ('placebo'/exp OR placebo) AND ('double blind procedure'/exp OR 'double blind procedure') AND ('relapse' OR 'recurrence'). In addition, reference lists of the included articles were manually searched for additional relevant published and unpublished research, including conference abstracts.

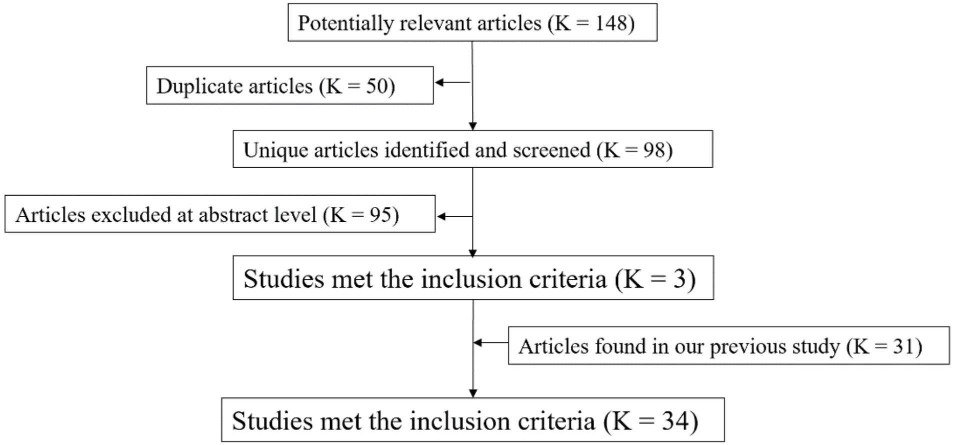

Articles included in the previous systematic review (K = 31)

1. Boulenger JP, Loft H, Florea I. A randomized clinical study of Lu AA21004 in the prevention of relapse in patients with major depressive disorder. J Psychopharmacol 2012; 26(11): 1408-1416.
2. Dalery J, Dagens-Lafont V, De Bodinat C. Efficacy of tianeptine vs placebo in the long-term treatment (16.5 months) of unipolar major recurrent depression*. Hum Psychopharmacol 2001; 16(S1): S39-S47.
3. Dekker J, Jonghe F, Tuynman H. The use of anti-depressants after recovery from depression. Eur J Psychiatry 2000; 14: 207-212.
4. Dobson KS, Hollon SD, Dimidjian S, Schmaling KB, Kohlenberg RJ, Gallop RJ et al. Randomized trial of behavioral activation, cognitive therapy, and antidepressant medication in the prevention of relapse and recurrence in major depression. J Consult Clin Psychol 2008; 76(3): 468-477.
5. Doogan DP, Caillard V. Sertraline in the prevention of depression. Br J Psychiatry 1992; 160: 217-222.
6. Feiger AD, Bielski RJ, Bremner J, Heiser JF, Trivedi M, Wilcox CS et al. Double-blind, placebo-substitution study of nefazodone in the prevention of relapse during continuation treatment of outpatients with major depression. Int Clin Psychopharmacol 1999; 14(1): 19-28.
7. Gilaberte I, Montejo AL, de la Gandara J, Perez-Sola V, Bernardo M, Massana J et al. Fluoxetine in the prevention of depressive recurrences: a double-blind study. J Clin Psychopharmacol 2001; 21(4): 417-424.
8. Goodwin GM, Boyer P, Emsley R, Rouillon F, de Bodinat C. Is it time to shift to better characterization of patients in trials assessing novel antidepressants? An

example of two relapse prevention studies with agomelatine. Int Clin Psychopharmacol 2013; 28(1): 20-28.
9. Goodwin GM, Emsley R, Rembry S, Rouillon F, Agomelatine Study G. Agomelatine prevents relapse in patients with major depressive disorder without evidence of a discontinuation syndrome: a 24-week randomized, double-blind, placebo-controlled trial. J Clin Psychiatry 2009; 70(8): 1128-1137.
10. Hochstrasser B, Isaksen PM, Koponen H, Lauritzen L, Mahnert FA, Rouillon F et al. Prophylactic effect of citalopram in unipolar, recurrent depression: placebo-controlled study of maintenance therapy. Br J Psychiatry 2001; 178: 304-310.
11. Keller MB, Kocsis JH, Thase ME, Gelenberg AJ, Rush AJ, Koran L et al. Maintenance phase efficacy of sertraline for chronic depression: a randomized controlled trial. JAMA 1998; 280(19): 1665-1672.
12. Kocsis JH, Thase ME, Trivedi MH, Shelton RC, Kornstein SG, Nemeroff CB et al. Prevention of recurrent episodes of depression with venlafaxine ER in a 1-year maintenance phase from the PREVENT Study. J Clin Psychiatry 2007; 68(7): 1014-1023.
13. McGrath PJ, Stewart JW, Quitkin FM, Chen Y, Alpert JE, Nierenberg AA et al. Predictors of relapse in a prospective study of fluoxetine treatment of major depression. Am J Psychiatry 2006; 163(9): 1542-1548.
14. Montgomery SA, Dunbar G. Paroxetine is better than placebo in relapse prevention and the prophylaxis of recurrent depression. Int Clin Psychopharmacol 1993; 8(3): 189-195.
15. Montgomery SA, Entsuah R, Hackett D, Kunz NR, Rudolph RL, Venlafaxine 335 Study G. Venlafaxine versus placebo in the preventive treatment of recurrent major depression. J Clin Psychiatry 2004; 65(3): 328-336.
16. Montgomery SA, Rasmussen JG, Tanghoj P. A 24-week study of 20 mg citalopram, 40 mg citalopram, and placebo in the prevention of relapse of major depression. Int Clin Psychopharmacol 1993; 8(3): 181-188.
17. Perahia DG, Gilaberte I, Wang F, Wiltse CG, Huckins SA, Clemens JW et al. Duloxetine in the prevention of relapse of major depressive disorder: double-blind placebo-controlled study. Br J Psychiatry 2006; 188: 346-353.
18. Perahia DG, Maina G, Thase ME, Spann ME, Wang F, Walker DJ et al. Duloxetine in the prevention of depressive recurrences: a randomized, double-blind, placebo-controlled trial. J Clin Psychiatry 2009; 70(5): 706-716.
19. Rapaport MH, Bose A, Zheng H. Escitalopram continuation treatment prevents relapse of depressive episodes. J Clin Psychiatry 2004; 65(1): 44-49.
20. Rickels K, Montgomery SA, Tourian KA, Guelfi JD, Pitrosky B, Padmanabhan SK et al. Desvenlafaxine for the prevention of relapse in major depressive disorder: results of a randomized trial. J Clin Psychopharmacol 2010; 30(1): 18-24.
21. Robert P, Montgomery SA. Citalopram in doses of 20-60 mg is effective in depression relapse prevention: a placebo-controlled 6 month study. Int Clin Psychopharmacol 1995; 10 Suppl 1: 29-35.

22. Rosenthal JZ, Boyer P, Vialet C, Hwang E, Tourian KA. Efficacy and safety of desvenlafaxine 50 mg/d for prevention of relapse in major depressive disorder:a randomized controlled trial. J Clin Psychiatry 2013; 74(2): 158-166.
23. Rouillon F, Warner B, Pezous N, Bisserbe JC. Milnacipran efficacy in the prevention of recurrent depression: a 12-month placebo-controlled study. Milnacipran recurrence prevention study group. Int Clin Psychopharmacol 2000; 15(3): 133-140.
24. Schmidt ME, Fava M, Robinson JM, Judge R. The efficacy and safety of a new enteric-coated formulation of fluoxetine given once weekly during the continuation treatment of major depressive disorder. J Clin Psychiatry 2000; 61(11): 851-857.
25. Shiovitz T, Greenberg WM, Chen C, Forero G, Gommoll CP. A Randomized, Double-blind, Placebo-controlled Trial of the Efficacy and Safety of Levomilnacipran ER 40-120mg/day for Prevention of Relapse in Patients with Major Depressive Disorder. Innov Clin Neurosci 2014; 11(1-2): 10-22.
26. Simon JS, Aguiar LM, Kunz NR, Lei D. Extended-release venlafaxine in relapse prevention for patients with major depressive disorder. J Psychiatr Res 2004; 38(3): 249-257.
27. Stein MK, Rickels K, Weise CC. Maintenance therapy with amitriptyline: a controlled trial. Am J Psychiatry 1980; 137(3): 370-371.
28. Terra JL, Montgomery SA. Fluvoxamine prevents recurrence of depression: results of a long-term, double-blind, placebo-controlled study. Int Clin Psychopharmacol 1998; 13(2): 55-62.
29. Thase ME, Nierenberg AA, Keller MB, Panagides J, Relapse Prevention Study G. Efficacy of mirtazapine for prevention of depressive relapse: a placebo-controlled double-blind trial of recently remitted high-risk patients. J Clin Psychiatry 2001; 62(10): 782-788.
30. Versiani M, Mehilane L, Gaszner P, Arnaud-Castiglioni R. Reboxetine, a unique selective NRI, prevents relapse and recurrence in long-term treatment of major depressive disorder. J Clin Psychiatry 1999; 60(6): 400-406.
31. Weihs KL, Houser TL, Batey SR, Ascher JA, Bolden-Watson C, Donahue RM et al. Continuation phase treatment with bupropion SR effectively decreases the risk for relapse of depression. Biol Psychiatry 2002; 51(9): 753-761.

Articles which we have found in the current literature search (K = 3)

1. Durgam S, Chen C, Migliore R, Prakash C, Thase ME. Relapse prevention with levomilnacipran ER in adults with major depressive disorder: A multicenter, randomized, double-blind, placebo-controlled study. Depress Anxiety 2019; 36(3): 225-234.
2. Durgam S, Gommoll C, Migliore R, Chen C, Chang CT, Aguirre M et al. Relapse prevention in adults with major depressive disorder treated with vilazodone: a randomized, double-blind, placebo-controlled trial. Int Clin Psychopharmacol 2018; 33(6): 304-311.
3. Thase ME, Jacobsen PL, Hanson E, Xu R, Tolkoff M, Murthy NV. Vortioxetine 5, 10, and 20 mg significantly reduces the risk of relapse compared with placebo in

patients with remitted major depressive disorder: The RESET study. J Affect Disord 2022; 303: 123-130.

Articles included in the previous systematic review but not in our study (K = 3)

1. Reimherr FW, Amsterdam JD, Quitkin FM, Rosenbaum JF, Fava M, Zajecka J et al. Optimal length of continuation therapy in depression: a prospective assessment during long-term fluoxetine treatment. Am J Psychiatry 1998; 155(9): 1247-1253.

Reasons for exclusion: the study included individuals with MDD as well as individuals with BD2.

2. Segal ZV, Bieling P, Young T, MacQueen G, Cooke R, Martin L et al. Antidepressant monotherapy vs sequential pharmacotherapy and mindfulness-based cognitive therapy, or placebo, for relapse prophylaxis in recurrent depression. Arch Gen Psychiatry 2010; 67(12): 1256-1264.

Reasons for exclusion: the antidepressant treatment arm included various antidepressants.

3. Stewart JW, Tricamo E, McGrath PJ, Quitkin FM. Prophylactic efficacy of phenelzine and imipramine in chronic atypical depression: likelihood of recurrence on discontinuation after 6 months' remission. Am J Psychiatry 1997; 154(1): 31-36.

Reasons for exclusion: the study included individuals with MDD as well as individuals with BD2.

Table S1. PRISMA for Network Meta-Analyses Checklist.

Section/Topic	Item #	Checklist Item	Reported on Page #
**TITLE**			
Title	1	Identify the report as a systematic review *incorporating a network meta-analysis (or related form of meta-analysis).*	1
**ABSTRACT**			
Structured summary	2	Provide a structured summary including, as applicable:<br>**Background:** main objectives<br>**Methods:** data sources; study eligibility criteria, participants, and interventions; study appraisal; and *synthesis methods, such as network meta-analysis.*<br>**Results:** number of studies and participants identified; summary estimates with corresponding confidence/credible intervals; *treatment rankings may also be discussed. Authors may choose to summarize pairwise comparisons against a chosen treatment included in their analyses for brevity*.<br>**Discussion/Conclusions:** limitations; conclusions and implications of findings.<br>**Other:** primary source of funding; systematic review registration number with registry name.	3-
**INTRODUCTION**			
Rationale	3	Describe the rationale for the review in the context of what is already known, *including mention of why a network meta-analysis has been conducted.*_	5-
Objectives	4	Provide an explicit statement of questions being addressed, with reference to participants, interventions, comparisons, outcomes, and study design (PICOS).	5-
**METHODS**			
Protocol and registration	5	Indicate whether a review protocol exists and if and where it can be accessed (e.g., Web address); and, if available, provide registration information, including registration number.	6-
Eligibility criteria	6	Specify study characteristics (e.g., PICOS, length of follow-up) and report characteristics (e.g., years considered, language, publication status) used as criteria for eligibility, giving rationale. *Clearly describe eligible treatments included in the*	6-

		*treatment network, and note whether any have been clustered or merged into the same node (with justification).*_	
Information sources	7	Describe all information sources (e.g., databases with dates of coverage, contact with study authors to identify additional studies) in the search and date last searched.	6-
Search	8	Present full electronic search strategy for at least one database, including any limits used, such that it could be repeated.	6-
Study selection	9	State the process for selecting studies (i.e., screening, eligibility, included in systematic review, and, if applicable, included in the meta-analysis).	6-
Data collection process	10	Describe method of data extraction from reports (e.g., piloted forms, independently, in duplicate) and any processes for obtaining and confirming data from investigators.	6-
Data items	11	List and define all variables for which data were sought (e.g., PICOS, funding sources) and any assumptions and simplifications made.	6-
**Geometry of the network**	**S1**	Describe methods used to explore the geometry of the treatment network under study and potential biases related to it. This should include how the evidence base has been graphically summarized for presentation, and what characteristics were compiled and used to describe the evidence base to readers.	6-
Risk of bias within individual studies	12	Describe methods used for assessing risk of bias of individual studies (including specification of whether this was done at the study or outcome level), and how this information is to be used in any data synthesis.	6-
Summary measures	13	State the principal summary measures (e.g., risk ratio, difference in means). *Also describe the use of additional summary measures assessed, such as treatment rankings and surface under the cumulative ranking curve (SUCRA)* values, as well as modified approaches used to present summary findings from meta-analyses.*	6-
Planned methods of analysis	14	Describe the methods of handling data and combining results of studies for each network meta-analysis. This should include, but not be limited to:<br>• *Handling of multi-arm trials;*<br>• *Selection of variance structure;*<br>• *Selection of prior distributions in Bayesian analyses; and*<br>• *Assessment of model fit.*	6-
**Assessment of Inconsistency**	**S2**	Describe the statistical methods used to evaluate the agreement of direct and indirect evidence in the treatment network(s) studied. Describe efforts taken to address its presence when found.	6-

Risk of bias across studies	15	Specify any assessment of risk of bias that may affect the cumulative evidence (e.g., publication bias, selective reporting within studies).	6-
Additional analyses	16	Describe methods of additional analyses if done, indicating which were pre-specified. This may include, but not be limited to, the following:<br>• Sensitivity or subgroup analyses;<br>• Meta-regression analyses;<br>• *Alternative formulations of the treatment network; and*<br>• *Use of alternative prior distributions for Bayesian analyses (if applicable).*_	6-
**RESULTS†**			
Study selection	17	Give numbers of studies screened, assessed for eligibility, and included in the review, with reasons for exclusions at each stage, ideally with a flow diagram.	10-
**Presentation of network structure**	**S3**	Provide a network graph of the included studies to enable visualization of the geometry of the treatment network.	10-
**Summary of network geometry**	**S4**	Provide a brief overview of characteristics of the treatment network. This may include commentary on the abundance of trials and randomized patients for the different interventions and pairwise comparisons in the network, gaps of evidence in the treatment network, and potential biases reflected by the network structure.	10-
Study characteristics	18	For each study, present characteristics for which data were extracted (e.g., study size, PICOS, follow-up period) and provide the citations.	10-
Risk of bias within studies	19	Present data on risk of bias of each study and, if available, any outcome level assessment.	10-
Results of individual studies	20	For all outcomes considered (benefits or harms), present, for each study: 1) simple summary data for each intervention group, and 2) effect estimates and confidence intervals. *Modified approaches may be needed to deal with information from larger networks.*	10-
Synthesis of results	21	Present results of each meta-analysis done, including confidence/credible intervals. *In larger networks, authors may focus on comparisons versus a particular comparator (e.g. placebo or standard care), with full findings presented in an*	10-

		appendix. League tables and forest plots may be considered to summarize pairwise comparisons. If additional summary measures were explored (such as treatment rankings), these should also be presented.	
**Exploration for inconsistency**	**S5**	Describe results from investigations of inconsistency. This may include such information as measures of model fit to compare consistency and inconsistency models, *P* values from statistical tests, or summary of inconsistency estimates from different parts of the treatment network.	10-
Risk of bias across studies	22	Present results of any assessment of risk of bias across studies for the evidence base being studied.	10-
Results of additional analyses	23	Give results of additional analyses, if done (e.g., sensitivity or subgroup analyses, meta-regression analyses, *alternative network geometries studied, alternative choice of prior distributions for Bayesian analyses*, and so forth).	10-
**DISCUSSION**			
Summary of evidence	24	Summarize the main findings, including the strength of evidence for each main outcome; consider their relevance to key groups (e.g., healthcare providers, users, and policy-makers).	13-
Limitations	25	Discuss limitations at study and outcome level (e.g., risk of bias), and at review level (e.g., incomplete retrieval of identified research, reporting bias). *Comment on the validity of the assumptions, such as transitivity and consistency. Comment on any concerns regarding network geometry (e.g., avoidance of certain comparisons).*	13-
Conclusions	26	Provide a general interpretation of the results in the context of other evidence, and implications for future research.	13-
**FUNDING**			16
Funding	27	Describe sources of funding for the systematic review and other support (e.g., supply of data); role of funders for the systematic review. This should also include information regarding whether funding has been received from manufacturers of treatments in the network and/or whether some of the authors are content experts with professional conflicts of interest that could affect use of treatments in the network.	

Table S2. The definition of relapse/recurrence.

Study name	The definition of relapse/recurrence
Stein 1980	No detailed information
Doogan 1992	CGI-S ≥ 4
Montgomery 1993 CIT	MADRS ≥ 22
Montgomery 1993 PAR	(1) CGI-S ≥ 4, (2) deterioration of CGI by ≥ 2 points, (3) met DSM-III-R criteria for MDD of 2 weeks, (4) needed antidepressant, or (5) Present of depressive symptomatology for > 7 days
Robert 1995	(1) MADRS ≥ 25 and (2) clinical judgment
Keller 1998	(1) met DSM-III-R criteria for MDD during ≥ 3 weeks, (2) CGI-S ≥ 4, (3) CGI-I ≥ 3, and (4) deterioration of HAMD24 by ≥ 4 points
Terra 1998	(1) met DSM-III-R criteria for MDD or (2) suicide attempt or completed suicide
Feiger 1999	(1) HAMD17 ≥ 18 for 2 consecutive visits or (2) lack of efficacy
Versiani 1999	(1) HAMD21 ≥ 18 or (2) deterioration of HAMD scores by ≥ 50%
Dekker 2000	HAMD17 ≥ 14
Rouillon 2000	(1) met DSM-III-R criteria for MDE and (2) HAMD21 ≥ 18 with the need to treat the recurrence
Schmidt 2000	(1) met SCID-P criteria for MDE and (2) deterioration of CGI-S by ≥ 2 points
Dalery 2001	(1) HAMD17 ≥ 15 and/or CGI ≥ 4 or (2) clinical judgement
Gilaberte 2001	(1) met DSM-III-R criteria for MDD, (2) HAMD17 ≥ 18, or (3) CGI ≥ 4
Hochstrasser 2001	MADRS ≥ 22
Thase 2001	Clinical judgment
Weihs 2002	The need for treatment intervention
Montgomery 2004	CGI-S ≥ 4
Rapaport 2004	(1) MADRS ≥ 22 or (2) withdrawal due to lack of efficacy
Simon 2004	(1) met DSM-IV criteria for MDD and (2) CGI-S ≥ 4 for 2 consecutive visits or final CGI-S ≥ 4
Perahia 2006	(1) deterioration of CGI-S by ≥ 2 points, (2) met MINI criteria for MDD for 2 consecutive visits
McGrath 2006	CGI-I ≥ 3 for 2 consecutive weeks

Kocsis 2007	(1) HAMD17 > 12 with a reduction of HAMD17 scores from acute phase by ≤ 50% for 2 consecutive visits, (2) withdrawal, and (3) met DSM-IV criteria for MDD
Dobson 2008	(1) HAMD17 ≥ 14 for 2 successive weeks, or (2) Psychiatric status rating ≥ 5 for 2 successive weeks
Goodwin 2009	(1) HAMD17 ≥ 16, (2) withdrawal due to lack of efficacy, or (3) suicide attempt or completed suicide
Perahia 2009	(1) CGI-S ≥ 4 and met DSM-IV criteria for MDD for ≥ 2 weeks, (2) met re-emergence criteria for 3 consecutive visits or 10 re-emergence visits, or (3) withdrawal due to lack of efficacy
Rickels 2010	(1) HAMD17 ≥ 16 or CGI-I ≥ 6, or (2) withdrawal due to lack of efficacy
Boulenger 2012	(1) MADRS ≥ 22, or (2) clinical judgment
Goodwin 2013	(1) HAMD17 ≥ 16, (2) withdrawal due to lack of efficacy, or (3) suicide attempt or completed suicide
Rosenthal 2013	(1) HAMD17 ≥ 16, (2) withdrawal due to lack of efficacy, (3) hospitalization for depression, or (4) suicide attempt or completed suicide
Shiovitz 2014	(1) MADRS ≥ 22 for 2 consecutive visits, (2) deterioration of CGI-I by ≥ 2 points for 2 consecutive visits, (3) withdrawal due to lack of efficacy, or (4) MADRS (item 10) ≥ 4
Durgam 2018	(1) MADRS ≥ 18 for 2 consecutive visits, (2) discontinuation due to lack of efficacy (needed medication switch and deterioration of CGI-S by ≥ 2 points), (3) hospitalization for depression
Durgam 2019	(1) deterioration of CGI-S by ≥ 2 points, (2) risk of suicide, (3) hospitalization for depression, (4) needed medication switch, (5) MADRS ≥ 18 for 2 consecutive visits
Thase 2022	1) MADRS ≥ 22, (2) lack of efficacy, (3) unsatisfactory treatment response

CGI: Clinical Global Impressions, CGI-I: Clinical Global Impression–Global Improvement, CGI-S: Clinical Global Impressions–severity of illness, DSM(R or TR): Diagnostic and Statistical Manual of Mental Disorders(Revision or Text Revision), HAMD: Hamilton Rating Scale for Depression, MADRS: Montgomery Åsberg Depression Rating Scale

Table S3. Data synthesis of 6-month relapse/recurrence.

Study name	6-month relapse rate
Stein 1980	Data at 26 weeks
Doogan 1992	Data at 26 weeks
Montgomery 1993 CIT	Data at 24 weeks
Montgomery 1993 PAR	Data at 26 weeks
Robert 1995	Data at 24 weeks
Keller 1998	Data at 26 weeks
Terra 1998	Data at 26 weeks
Feiger 1999	Data at 26 weeks
Versiani 1999	Data at 26 weeks
Dekker 2000	Data at 22 weeks
Rouillon 2000	Data at 26 weeks
Schmidt 2000	Data at 25 weeks
Dalery 2001	Data at 26 weeks
Gilaberte 2001	Data at 26 weeks
Hochstrasser 2001	Data at 26 weeks
Thase 2001	Data at 26 weeks
Weihs 2002	Data at 26 weeks
Montgomery 2004	Data at 26 weeks
Rapaport 2004	Data at 26 weeks
Simon 2004	Data at 26 weeks
Perahia 2006	Data at 26 weeks
McGrath 2006	Data at 26 weeks
Kocsis 2007	Data at 26 weeks

Dobson 2008	Data at 26 weeks
Goodwin 2009	Data at 24 weeks
Perahia 2009	Data at 26 weeks
Rickels 2010	Data at 26 weeks
Boulenger 2012	Data at 26 weeks
Goodwin 2013	Data at 26 weeks
Rosenthal 2013	Data at 26 weeks
Shiovitz 2014	Data at 24 weeks
Durgam 2018	Data at 26 weeks
Durgam 2019	Data at 26 weeks
Thase 2022	Data at 26 weeks

Table S4. Study characteristics.

Study name	Region	AD	Sponsor	PT status	Diagnosis	Total n	Mean age±SD	Female (%)	Number of episodes	Duration of preliminary phase (w)	Duration of RCT phase (w)	Mean score at baseline of acute study	AD dose	Mean final dose (mg/d)	Dosing schedule	Discontinuation method
Stein 1980	USA	AMI	Academia	OP	DSM3	55	42.3±12.8	65	NI	8	26	HAMD: 25.1**	100-150	NI	Flexible	AB
Doogan 1992	International	SER	Industry	OP	DSM3	300	51	69	NI	8	44	HAMD17: ≥ 17	50-200	69.3-82.1	Flexible	AB
Montgomery 1993a	UK	PAR	Industry	NI	DSM3R	135	47.09±8.76	78.52	NI	8	52	HAMD21: 26.9	20-30	NI	Flexible	NI
Montgomery 1993b	International	CIT	Industry	Both	DSM3R	147	NI	NI	NI	6	24	MADRS: ≥ 22	20 or 40	30.86	Fixed	NI
Robert 1995	France	CIT	Industry	NI	DSM3R	226	NI	71.68	NI	8	24	MADRS: ≥ 25	20, 40 or 60	NI	Fixed	NI
Keller 1998	USA	SER	Industry	OP	DSM3R	161	41.63±9.38	65.84	1.85	28	76	HAMD24: 24.9	50-200	146.1	Flexible	TAP
Terra 1998	France	FLUV	Industry	NI	DSM3R	204	44.73±11.00	73.53	3.5	24	52	MADRS: ≥ 24	100	100	Fixed	NI
Feiger 1999	USA	NEF	Industry	OP	DSM3R	131	41.31±10.98	71.76	1.60	16	36	HAMD: 24.3	100-600	412	Flexible	NI
Versiani 1999	International	REB	Industry	Both	DSM3R	286	42.86±11.89	73.43	NI	6	46	HAMD21: 29.6	4-8	NI	Flexible	NI
Dekker 2000	Netherland	FLUO	Industry	OP	DSM3R	30	37±10	61.9	NI	16	22	HAMD17: ≥ 14	20	20	Fixed	NI
Rouillon 2000	France	MIL	Industry	Both	DSM3R	214	45.33±10.1	67.28	2.98	26	52	HAMD21: 25.1	100	100	Fixed	NI
Schmidt 2000	USA	FLUO*	Industry	OP	DSM4	501	41.47±11.34	68.26	NI	13	25	HAMD17: ≥ 18	20	20	Fixed	AB
Dalery 2001	France	TIA	Industry	Both	DSM3R	185	43.31±	65.41	2.56	6	79	HAMD17: 23.3	37.5	37.5	Fixed	NI

Gilaberte 2001	Spain	FLUO	Industry	OP	DSM3R	140	44.1	78.6	2.45	32	52	HAMD17: 24	20	20	Fixed	NI
Hochstrasser 2001	International	CIT	Industry	Both	DSM4	269	43.1±10.64	71.2	3.5	22-25	48-78 w	MADRS: 30.5	20, 40 or 60	33.94	Fixed	NI
Thase 2001	USA	MIR	Industry	NI	DSM4	161	40.41±11.61	50.64	NI	8-12	40	HAMD17: 22.7	30-45	38.6	Flexible	AB
Weihs 2002	USA	BUP	Industry	NI	DSM4	423	39.65±0.25	65.01	3.00	8	44	HAMD21: ≥ 18	300	290	Fixed	NI
Montgomery 2004	International	VEN	Industry	OP	DSM3R	235	43.65±11.08	68.89	3.21	26	52	HAMD21: 25.2	100~200	132-152***	Flexible	TAP
Rapaport 2004	USA	ESC	Industry	OP	DSM4	274	42.53±11.69	60.95	NI	8	36	MADRS: ≥22	10 or 20	NI	Fixed	NI
Simon 2004	NA	VEN	Industry	NI	DSM4	318	42.05	64.38	NI	8	26	HAMD21: 24.5	75, 150 or 225	177–191	Fixed	TAP
Perahia 2006	International	DUL	Industry	NI	DSM4	278	45.24±12.25	72.66	NI	12	26	HAMD17: 23.7	60	60	Fixed	TAP
McGrath 2006	USA	FLUO	Academia	NI	DSM4	262	38.2±10.9	55.3	NI	12	52	HAMD17: 17.7****	40 or 60	45.8	Fixed	NI
Kocsis 2007	USA	VEN	Industry	OP	DSM4	267	42.3	68	NI	36	52	HAMD17: 22.4	75-300	220.8	Flexible	TAP
Dobson 2008	USA	PAR	Academia	OP	DSM4	49	38.93±10.04	78.2	1.12	16	52	HAMD17: 20.9	10-50	NI	Flexible	TAP
Goodwin 2009	International	AGO	Industry	OP	DSM4TR	339	43.25±10.58	74.31	3.6	8 or 10	24	HAMD17: 27.0	25 or 50	NI	Fixed	AB
Perahia 2009	International	DUL	Industry	OP	DSM4	288	47.54±12.54	71.53	4.2	34	52	HAMD17: 23.1	60-120	84.3	Fixed	TAP
Rickels 2010	International	DES	Industry	OP	DSM4	375	42.75±12.04	67.47	NI	12	26	HAMD17: 24.2	200-400	NI	Fixed	TAP

Boulenger 2012	International	VOR	Industry	Both	DSM4TR	400	44.95±12.24	63.13	2.1	12	24-64	MADRS: 32.3	5 or 10	8.53	Fixed	AB
Goodwin 2013	International	AGO	Industry	OP	DSM4TR	367	45.64±10.3	77.92	4.4	8	42	HAMD17: 26.3	25	25	Fixed	NI
Rosenthal 2013	International	DESV	Industry	OP	DSM4	548	45.95 ±13	71.35	2.12	20	26	HAMD17: 24.2	50	50	Fixed	TAP
Shiovitz 2014	USA and Canada	LEV	Industry	OP	DSM4TR	348	43.28±12.25	57.97	4.77	12	24	MADRS: 30.7	40, 80 or 120	79	Fixed	TAP
Durgam 2018	International	VIL	Industry	OP	DSM4TR	564	45.25±12.21	63.06	4.60	20	28	MADRS: 31.7	20 or 40	30.05	Fixed	TAP
Durgam 2019	USA	LEV	Industry	OP	DSM5	324	45.39±13.46	67.28	5.2	20	26	MADRS: 32.2	40-120	NI	Fixed	TAP
Thase 2022	USA	VOR	Industry	OP	DSM4TR	580	45.1±13.23	72.42	NI	16	28	MADRS: 33.9	5, 10 or 20	11.72	Fixed	AB

AB: abrupt discontinuation, AD: antidepressant, AGO: agomelatine, AMI: amitriptyline, Both: both outpatient and inpatient, BUP: bupropion, CGI-S: Clinical Global Impressions - severity of illness, CIT: citalopram, d: day, DES: desvenlafaxine, DSM(R or TR): Diagnostic and Statistical Manual of Mental Disorders(Revision or Text Revision), DUL: duloxetine, ESC: escitalopram, FLUO: fluoxetine, FLUV: fluvoxamine, HAMD: Hamilton Rating Scale for Depression, LEV: levomilnacipran, MADRS: Montgomery Åsberg Depression Rating Scale, MIL: milnacipran, MIR: mirtazapine, n: number of patients, NEF: nefazodone, NI: not information, OP: outpatient, PAR: paroxetine, PT: patient, RCT: randomized controlled trial, REB: reboxetine, SD: standard deviation, SER: sertraline, TAP: tapering discontinuation, TIA: tianeptine, UK: United Kingdom, USA: United States of America, VEN: venlafaxine, VIL: vilazodone, VOR: vortioxetine, w: week

* daily or once weekly

** This study did not report the detailed information that participants in acute study had a requirement of a scale-derived minimum of symptoms at baseline.

*** The dose was the mean dose during the study.

**** This study reported that participants in acute study did not have a requirement of a scale-derived minimum of symptoms at baseline.

Table S5. Transitivity assessment.

<table>
<tr><th></th><th>Boxplot</th><th>Kruskal–Wallis equality of populations rank test for continuous variables or the Pearson chi-squared test for binary and categorical variables (or the Fisher exact test whether more than 20% of cells had an expected frequency below 5)</th></tr>
<tr><td>Mean age (K = 32)</td><td>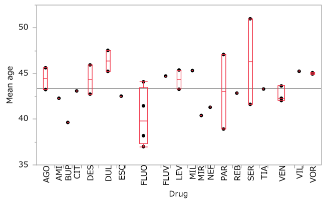
</td><td>Chi-squared with ties = 22.06 (df = 19), p = 0.2812</td></tr>
<tr><td>Proportion of females (K = 33)</td><td>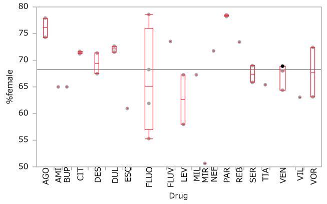
</td><td>Chi-squared with ties = 17.19 (df = 19), p = 0.5767</td></tr>
<tr><td>Number of episodes (K = 18)</td><td>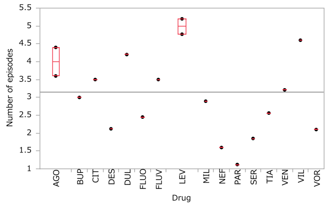
</td><td>Chi-squared with ties = 16.91 (df = 15), p = 0.3241</td></tr>
<tr><td>Total number of participants (K= 34)</td><td>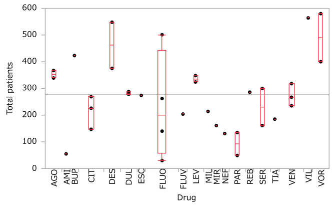
</td><td>Chi-squared with ties = 25.55 (df = 19), p = 0.1431</td></tr>
</table>

Patient status (K= 26)	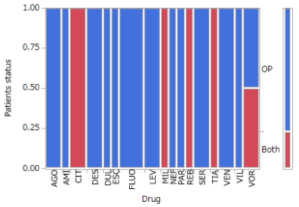	Fisher $chi^2 = 22.18$, $p = 0.1089$
Publication year (K= 34)	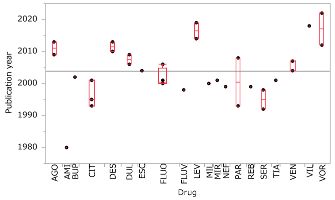	Chi-squared with ties = 28.80 (df = 19), p = 0.0692
Sponsorship (K= 34)	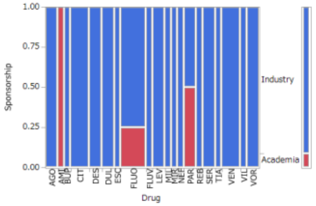	Fisher $chi^2 = 18.462$, $p = 0.4918$
Duration of preliminary phase (K= 34)	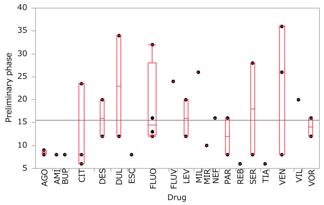	Chi-squared with ties = 17.41 (df = 19), p = 0.5624
Country (K =33)	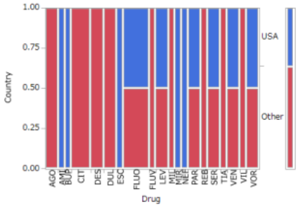	Fisher $chi^2 = 17.88$, $p = 0.5308$

Risk of bias (K= 34)	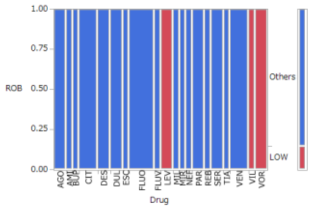	Fisher chi^2 = 34.00, p = 0.0184
Discontinuation method (K = 19)	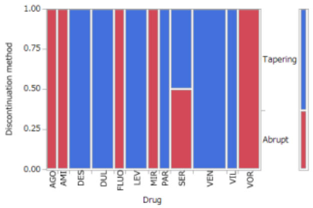	Fisher chi^2 = 16.85, p = 0.1124
Dosage schedule (K= 34)	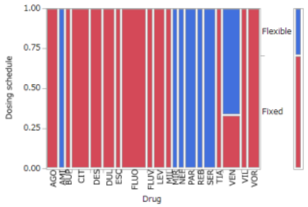	Fisher chi^2 = 0.0426, p = 0.0726
Antidepressant dose (K= 31)	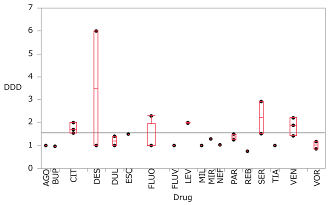	Chi-squared with ties = 19.81 (df = 17), p = 0.2839

Table S6. Risk of bias summary.

	Randomization process	Deviation from intended intervention	Missing outcome data	Measurement of the outcome	Selection of the reported result	Overall risk of bias
Stein 1980	Some concerns	Some concerns	Low	Some concerns	Low	Some concerns
Doogan 1992	Some concerns	Low	Low	Some concerns	Low	Some concerns
Montgomery 1993a PAR	Some concerns	Some concerns	Low	Some concerns	Low	Some concerns
Montgomery 1993b CIT	Some concerns	Some concerns	Low	Some concerns	Low	Some concerns
Robert 1995	Some concerns	Some concerns	Low	Some concerns	Low	Some concerns
Keller 1998	Some concerns	Low	Low	Low	Low	Some concerns
Terra 1998	Some concerns	Some concerns	Low	Some concerns	Low	Some concerns
Feiger 1999	Some concerns	Low	Low	Some concerns	Low	Some concerns
Versiani 1999	Some concerns	Some concerns	Low	Some concerns	Low	Some concerns
Dekker 2000	Some concerns*	Low	Low	Low	Low	Some concerns
Rouillon 2000	Some concerns	Some concerns	Low	Some concerns	Low	Some concerns
Schmidt 2000	Some concerns	Some concerns	Low	Some concerns	Low	Some concerns
Dalery 2001	Some concerns	Low	Low	Some concerns	Low	Some concerns
Gilaberte 2001	Some concerns	Some concerns	Low	Some concerns	Low	Some concerns
Hochstrasser 2001	Some concerns	Some concerns	Low	Some concerns	Low	Some concerns
Thase 2001	Some concerns	Low	Low	Some concerns	Low	Some concerns
Weihs 2002	Some concerns	Low	Low	Some concerns	Low	Some concerns
Montgomery 2004	Some concerns	Some concerns	Low	Some concerns	Low	Some concerns
Rapaport 2004	Some concerns	Some concerns	Low	Some concerns	Low	Some concerns
Simon 2004	Some concerns	Some concerns	Low	Some concerns	Low	Some concerns
Perahia 2006	Some concerns	Some concerns	Low	Some concerns	Low	Some concerns
McGrath 2006	Some concerns*	Low	Low	Low	Low	Some concerns

Kocsis 2007	Some concerns	Low	Low	Low	Low	Some concerns
Dobson 2008	Some concerns*	Some concerns	Low	Some concerns	Low	Some concerns
Goodwin 2009	Some concerns*	Low	Low	Low	Low	Some concerns
Perahia 2009	Some concerns	Some concerns	Low	Some concerns	Low	Some concerns
Rickels 2010	Some concerns	Some concerns	Low	Some concerns	Low	Some concerns
Boulenger 2012	Low	Low	Low	Low	Low	Low
Goodwin 2013	Some concerns*	Low	Low	Low	Low	Some concerns
Rosenthal 2013	Some concerns*	Some concerns	Low	Some concerns	Low	Some concerns
Shiovitz 2014	Low	Low	Low	Low	Low	Low
Durgam 2018	Low	Low	Low	Low	Low	Low
Durgam 2019	Low	Low	Low	Low	Low	Low
Thase 2022	Low	Low	Low	Low	Low	Low

* We did not find the sufficient information to assess the risk of bias with respect to “allocation concealment.”

“Some concerns” in each domain: because we did not find the sufficient information to assess the risk of bias with respect to the domains.

Risk of bias in RCTs for the main outcomes was assessed independently using the Cochrane risk-of-bias tool for randomized trials (RoB 2).[2]
https://www.riskofbias.info/welcome

Appendix S1. 6-month relapse rate. (K = 34, n = 9189).

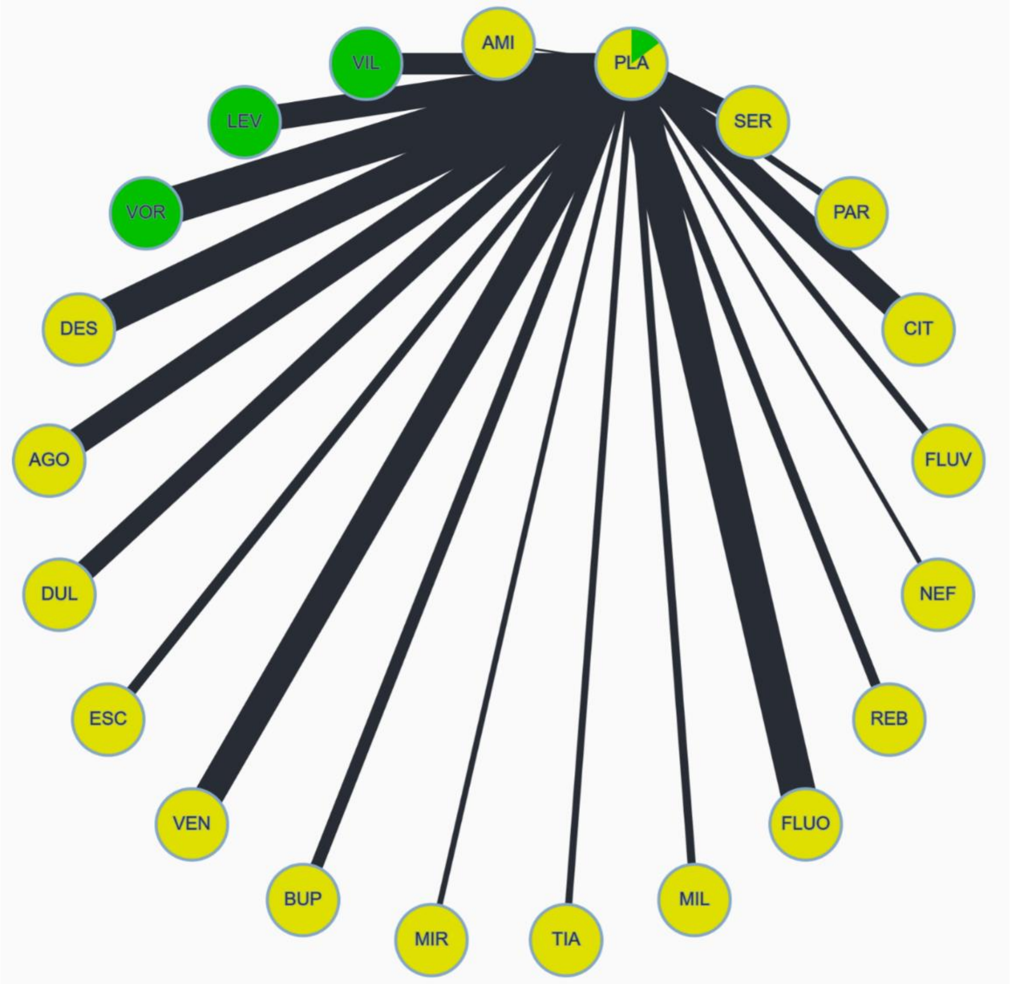

Node color by risk of bias

Green: low overall risk of bias

Yellow: moderate overall risk of bias

Edge width by sample size

League table (risk ratio with 95% credible interval)

PLA																				
0.693 (0.449, 1.084)	AGO																			
**0.393 (0.161, 0.852)**	0.563 (0.212, 1.360)	AMI																		
0.728 (0.418, 1.288)	1.051 (0.515, 2.096)	1.862 (0.713, 5.373)	BUP																	
**0.396 (0.260, 0.598)**	0.572 (0.309, 1.044)	1.013 (0.417, 2.686)	0.544 (0.270, 1.087)	CIT																
**0.527 (0.347, 0.787)**	0.763 (0.412, 1.414)	1.354 (0.559, 3.548)	0.723 (0.357, 1.432)	1.328 (0.729, 2.430)	DES															
**0.447 (0.273, 0.719)**	0.643 (0.326, 1.224)	1.143 (0.440, 3.072)	0.613 (0.288, 1.270)	1.124 (0.586, 2.122)	0.844 (0.438, 1.577)	DUL														
0.605 (0.326, 1.142)	0.878 (0.407, 1.875)	1.539 (0.575, 4.582)	0.832 (0.356, 1.939)	1.533 (0.731, 3.263)	1.148 (0.547, 2.469)	1.354 (0.616, 3.062)	ESC													
**0.583 (0.410, 0.789)**	0.843 (0.470, 1.393)	1.484 (0.612, 3.831)	0.800 (0.408, 1.483)	1.471 (0.855, 2.480)	1.113 (0.644, 1.820)	1.309 (0.716, 2.299)	0.963 (0.462, 1.900)	FLUO												
**0.298 (0.114, 0.686)**	0.426 (0.150, 1.113)	0.763 (0.225, 2.566)	0.409 (0.132, 1.097)	0.748 (0.266, 1.908)	0.564 (0.197, 1.428)	0.667 (0.229, 1.744)	0.491 (0.155, 1.399)	0.513 (0.190, 1.256)	FLUV											
0.560 (0.305, 1.020)	0.919 (0.481, 1.739)	1.630 (0.628, 4.463)	0.872 (0.423, 1.795)	1.613 (0.852, 3.018)	1.210 (0.658, 2.270)	1.429 (0.730, 2.870)	1.051 (0.476, 2.261)	1.096 (0.627, 1.984)	2.129 (0.826, 6.283)	LEV										
0.719 (0.331, 1.545)	1.029 (0.427, 2.630)	1.856 (0.615, 5.939)	0.988 (0.373, 2.456)	1.806 (0.758, 4.475)	1.371 (0.566, 3.240)	1.618 (0.638, 4.081)	1.191 (0.434, 3.212)	1.234 (0.539, 2.863)	2.424 (0.760, 8.416)	1.127 (0.467, 2.756)	MIL									

**0.402 (0.190, 0.829)**	0.581 (0.246, 1.315)	1.029 (0.346, 3.263)	0.554 (0.217, 1.374)	1.014 (0.430, 2.334)	0.760 (0.329, 1.762)	0.910 (0.378, 2.190)	0.665 (0.254, 1.718)	0.691 (0.306, 1.562)	1.347 (0.435, 4.527)	0.631 (0.268, 1.485)	0.554 (0.196, 1.648)	MIR								
**0.149 (0.018, 0.610)**	**0.215 (0.025, 0.955)**	0.378 (0.041, 1.963)	**0.205 (0.024, 0.920)**	0.376 (0.045, 1.644)	0.281 (0.033, 1.225)	0.337 (0.039, 1.508)	0.246 (0.029, 1.148)	0.257 (0.032, 1.107)	0.503 (0.054, 2.681)	0.234 (0.027, 1.017)	0.203 (0.022, 1.064)	0.373 (0.041, 1.791)	NEF							
**0.416 (0.220, 0.759)**	0.601 (0.279, 1.250)	1.056 (0.396, 3.086)	0.568 (0.241, 1.290)	1.052 (0.499, 2.164)	0.789 (0.372, 1.625)	0.936 (0.424, 2.020)	0.681 (0.285, 1.645)	0.715 (0.353, 1.445)	1.401 (0.490, 4.344)	0.651 (0.302, 1.381)	0.576 (0.210, 1.553)	1.029 (0.388, 2.711)	2.777 (0.592, 24.878)	PAR						
**0.520 (0.278, 0.968)**	0.754 (0.344, 1.578)	1.338 (0.488, 3.868)	0.715 (0.306, 1.655)	1.318 (0.614, 2.804)	0.988 (0.467, 2.099)	1.164 (0.521, 2.636)	0.859 (0.353, 2.070)	0.896 (0.455, 1.857)	1.752 (0.622, 5.553)	0.815 (0.378, 1.790)	0.727 (0.267, 1.995)	1.285 (0.493, 3.403)	3.483 (0.731, 30.248)	1.251 (0.526, 2.991)	REB					
**0.165 (0.083, 0.305)**	**0.237 (0.107, 0.502)**	0.415 (0.151, 1.267)	**0.225 (0.094, 0.529)**	**0.416 (0.186, 0.890)**	**0.310 (0.142, 0.670)**	**0.368 (0.163, 0.838)**	**0.271 (0.108, 0.673)**	**0.282 (0.134, 0.587)**	0.553 (0.188, 1.781)	**0.258 (0.112, 0.566)**	**0.228 (0.082, 0.632)**	0.407 (0.154, 1.067)	1.098 (0.227, 9.845)	**0.397 (0.161, 0.973)**	**0.317 (0.127, 0.773)**	SER				
**0.258 (0.084, 0.698)**	0.372 (0.111, 1.080)	0.668 (0.170, 2.502)	0.357 (0.103, 1.063)	0.653 (0.194, 1.923)	0.494 (0.148, 1.451)	0.580 (0.172, 1.761)	0.425 (0.116, 1.370)	0.446 (0.140, 1.283)	0.868 (0.221, 3.473)	0.407 (0.120, 1.212)	0.359 (0.091, 1.247)	0.647 (0.174, 2.188)	1.748 (0.283, 17.388)	0.625 (0.174, 2.068)	0.499 (0.139, 1.626)	1.564 (0.440, 5.219)	TIA			
**0.555 (0.386, 0.784)**	0.800 (0.457, 1.374)	1.425 (0.592, 3.565)	0.758 (0.386, 1.488)	1.405 (0.808, 2.374)	1.055 (0.599, 1.795)	1.242 (0.690, 2.291)	0.917 (0.437, 1.865)	0.951 (0.594, 1.563)	1.854 (0.749, 5.084)	0.869 (0.476, 1.569)	0.774 (0.328, 1.804)	1.383 (0.620, 3.131)	3.762 (0.859, 32.110)	1.330 (0.665, 2.818)	1.063 (0.514, 2.162)	**3.381 (1.625, 7.259)**	2.134 (0.739, 6.943)	VEN		
0.990 (0.512, 1.934)	1.430 (0.650, 3.122)	2.518 (0.927, 7.507)	1.358 (0.569, 3.207)	**2.485 (1.136, 5.494)**	1.874 (0.850, 4.201)	2.218 (0.984, 5.124)	1.624 (0.678, 4.049)	1.696 (0.835, 3.671)	**3.339 (1.148, 10.778)**	1.548 (0.708, 3.594)	1.375 (0.482, 3.845)	2.454 (0.909, 6.888)	**6.726 (1.432, 63.135)**	2.368 (0.982, 6.186)	1.905 (0.760, 4.867)	**6.005 (2.466, 15.812)**	**3.840 (1.149, 14.142)**	1.774 (0.852, 3.852)	VIL	
**0.518 (0.335, 0.799)**	0.743 (0.401, 1.371)	1.312 (0.532, 3.543)	0.708 (0.348, 1.432)	1.298 (0.717, 2.428)	0.977 (0.546, 1.771)	1.150 (0.621, 2.295)	0.853 (0.398, 1.871)	0.885 (0.531, 1.559)	1.743 (0.683, 4.919)	0.807 (0.422, 1.530)	0.717 (0.299, 1.730)	1.276 (0.551, 3.023)	3.438 (0.781, 28.458)	1.250 (0.596, 2.678)	0.995 (0.467, 2.163)	**3.131 (1.456, 6.876)**	2.003 (0.684, 6.677)	0.930 (0.544, 1.615)	0.519 (0.232, 1.153)	VOR

Evaluation of heterogeneity

Network meta-analysis

Global heterogeneity was assessed by means of τ^2 (low: $\tau^2 \leq 0.010$; moderate: $0.010 < \tau^2 \leq 0.242$; high: $\tau^2 > 0.242$).

Huhn M, et al. Lancet 2019;394(10202):939-51

Rhodes KM, et al. J Clin Epidemiol 2015;68(1):52-60

Between study variance (τ^2): 0.044

Heterogeneity assessment: Moderate

Pairwise meta-analysis

Local heterogeneity was assessed by means of I^2.

0% to 40%: might not be important, 30% to 60%: may represent moderate heterogeneity, 50% to 90%: may represent substantial heterogeneity, and 75% to 100%: considerable heterogeneity.

	Risk ratio (95% confidence interval)	I^2
AGO vs PLA	**0.699 (0.491, 0.996)**	89.0%
AMI vs PLA	**0.398 (0.191, 0.833)**	na
BUP vs PLA	0.725 (0.466, 1.127)	na
CIT vs PLA	**0.398 (0.273, 0.580)**	51.4%
DES vs PLA	**0.526 (0.376, 0.736)**	2.0%
DUL vs PLA	**0.451 (0.297, 0.685)**	0.0%
ESC vs PLA	0.604 (0.357, 1.023)	na
FLUO vs PLA	**0.585 (0.445, 0.771)**	0.0%
FLUV vs PLA	**0.300 (0.130, 0.693)**	na
LEV vs PLA	**0.632 (0.419, 0.953)**	0.0%
MIL vs PLA	0.721 (0.358, 1.452)	na

MIR vs PLA	**0.409 (0.215, 0.778)**	na
NEF vs PLA	**0.169 (0.038, 0.759)**	na
PAR vs PLA	**0.445 (0.249, 0.794)**	66.9%
REB vs PLA	**0.523 (0.310, 0.884)**	na
SER vs PLA	**0.170 (0.093, 0.309)**	0.0%
TIA vs PLA	**0.267 (0.101, 0.703)**	na
VEN vs PLA	**0.560 (0.415, 0.755)**	29.5%
VIL vs PLA	0.983 (0.552, 1.752)	na
VOR vs PLA	**0.516 (0.358, 0.743)**	0.0%

Forest plot (vs placebo, the numbers are risk ratios with 95% confidence interval)

Treatments are ranked according to their SUCRA.

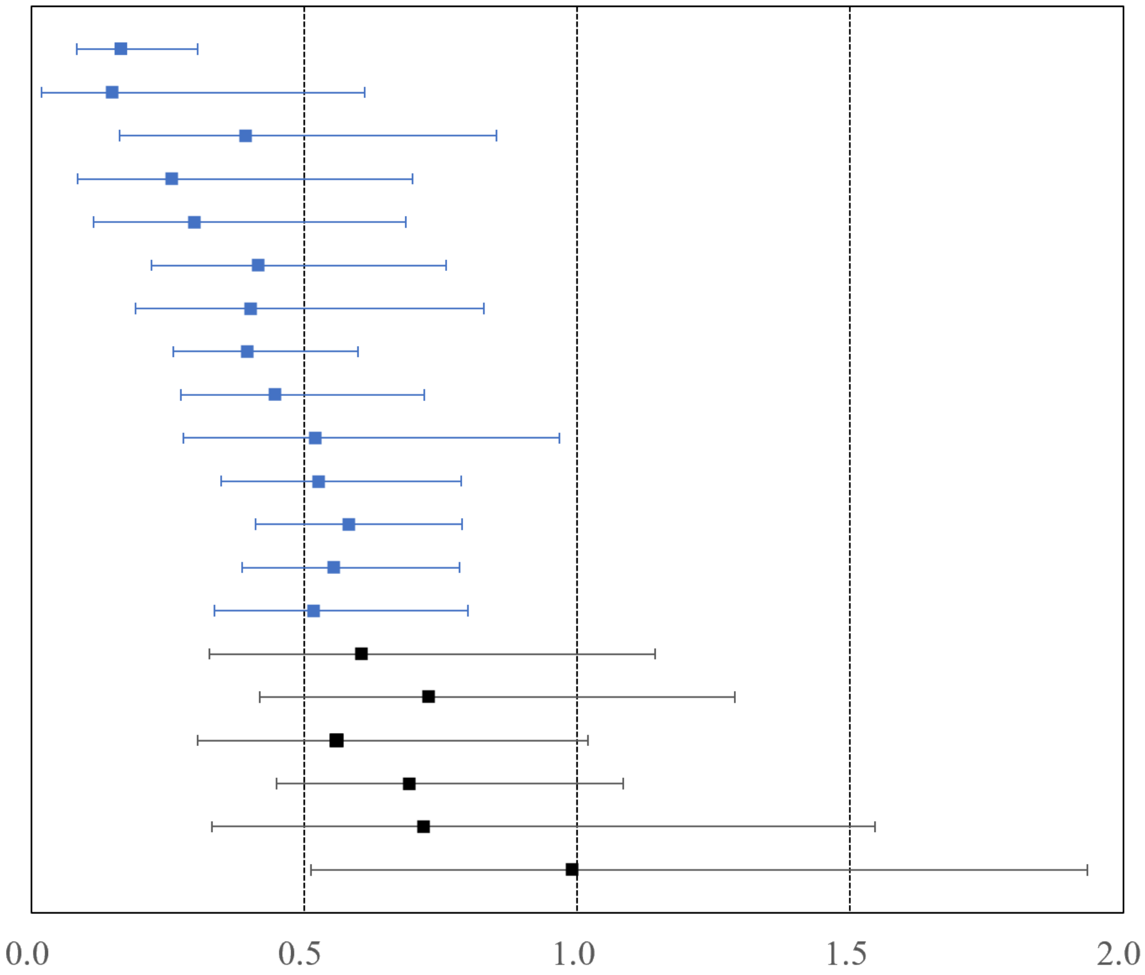

Drug	RR (95% CrI)
SER	0.165 (0.083, 0.305)
NEF	0.149 (0.018, 0.610)
AMI	0.393 (0.161, 0.852)
TIA	0.258 (0.084, 0.698)
FLUV	0.298 (0.114, 0.686)
PAR	0.416 (0.220, 0.759)
MIR	0.402 (0.190, 0.829)
CIT	0.396 (0.260, 0.598)
DUL	0.447 (0.273, 0.719)
REB	0.520 (0.278, 0.968)
DES	0.527 (0.347, 0.787)
FLUO	0.583 (0.410, 0.789)
VEN	0.555 (0.386, 0.784)
VOR	0.518 (0.335, 0.799)
ESC	0.605 (0.326, 1.142)
BUP	0.728 (0.418, 1.288)
LEV	0.560 (0.305, 1.020)
AGO	0.693 (0.449, 1.084)
MIL	0.719 (0.331, 1.545)
VIL	0.990 (0.512, 1.934)

Funnel plot (only double-blind, placebo-controlled trials)

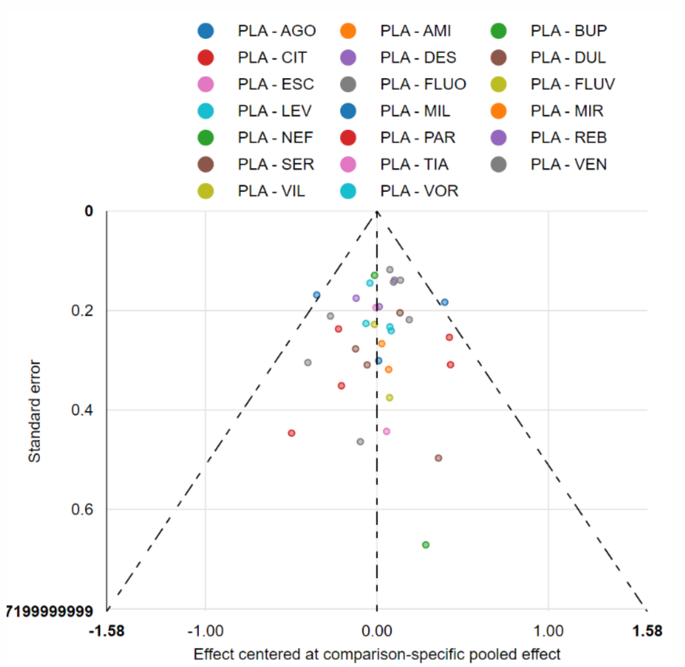

Meta-regression analysis (the placebo was the control)

	τ^2	β , median (95% CrI)
Mean age (K = 32)	0.035	-0.302 (-0.716, 0.084)
Proportion of females (K = 33)	0.052	-0.190 (-0.651, 0.251)
Number of episodes (K = 18)	0.211	1.007 (-2.121, 3.922)
Total number of participants (K= 34)	0.054	0.082 (-0.409, 0.553)
Patient status (K= 26)	0.070	-0.096 (-1.031, 0.865)
Publication year (K= 34)	0.051	0.347 (-0.365, 1.175)
Sponsorship (K= 34)	0.048	0.338 (-0.200, 0.992)
Duration of preliminary phase (K= 34)	0.037	-0.168 (-0.473, 0.146)
Country (K =33)	0.050	0.272 (-0.137, 0.701)
Discontinuation method (K = 19)	0.012	-0.200 (-1.269, 1.181)
Risk of bias (K= 34)	0.045	-0.352 (-4.819, 2.960)
Antidepressant class (K= 34)	0.045	-0.482 (-6.895, 3.552)
Dosage schedule (K= 34)	0.052	-0.160 (-0.874, 0.548)
Antidepressant dose (K= 31)	0.012	0.132 (-0.090, 0.380)

τ^2 value of the primary analysis was 0.044.

CINeMA confidence rating

CINeMA is a web application that simplifies the evaluation of confidence in the findings from a network meta-analysis. CINeMA is based on a methodological framework described in the following articles, which consider the following six domains: within-study bias, reporting bias, indirectness, imprecision, heterogeneity, and incoherence. CINeMA grades the confidence in the results of each treatment comparison as high, moderate, low, or very low.

Nikolakopoulou A, et al., PLOS Medicine 2020 17 1-19

Papakonstantinou T, et al., Campbell Systematic Reviews 2020 16 e1080

(1) Within-study bias: Risk of bias in RCTs for the main outcomes was assessed independently using the Cochrane risk-of-bias tool for randomized trials (RoB 2).
https://www.riskofbias.info/welcome

(2) Reporting bias: Comparison-adjusted funnel plots with less than 10 studies are not meaningful. Therefore, all comparisons were "Suspected."

(3) Indirectness: No indirectness was assumed. Selected rule: Average

(4) Imprecision: For placebo comparisons the clinically meaningful threshold was set at a risk ratio of higher or lower than 1. For comparisons of two antidepressants the clinically meaningful threshold was set at risk ratio of 0.8 and 1.25.

(5) Heterogeneity: We used recommendations automatically provided by CINeMA.

(6) Incoherence: We used recommendations automatically provided by CINeMA. If the comparison had only indirect evidence, the comparison was downgraded one level.

Comparison	Number of studies	Within-study bias	Reporting bias	Indirectness	Imprecision	Heterogeneity	Incoherence	Confidence rating
AGO vs PLA	2	Some concerns	Some concerns	No concerns	Major concerns	Major concerns	Major concerns	Very low
AMI vs PLA	1	Some concerns	Some concerns	No concerns	No concerns	No concerns	Major concerns	Very low
BUP vs PLA	1	Some concerns	Some concerns	No concerns	Major concerns	No concerns	Major concerns	Very low
CIT vs PLA	3	Some concerns	Some concerns	No concerns	No concerns	No concerns	Major concerns	Very low
DES vs PLA	2	Some concerns	Some concerns	No concerns	No concerns	No concerns	Major concerns	Very low
DUL vs PLA	2	Some concerns	Some concerns	No concerns	No concerns	No concerns	Major concerns	Very low
ESC vs PLA	1	Some concerns	Some concerns	No concerns	Major concerns	No concerns	Major concerns	Very low
FLUO vs PLA	4	Some concerns	Some concerns	No concerns	Major concerns	No concerns	Major concerns	Very low
FLUV vs PLA	1	Some concerns	Some concerns	No concerns	No concerns	No concerns	Major concerns	Very low
LEV vs PLA	2	No concerns	Some concerns	No concerns	Major concerns	Major concerns	Major concerns	Very low
MIL vs PLA	1	Some concerns	Some concerns	No concerns	Major concerns	No concerns	Major concerns	Very low
MIR vs PLA	1	Some concerns	Some concerns	No concerns	No concerns	No concerns	Major concerns	Very low
NEF vs PLA	1	Some concerns	Some concerns	No concerns	No concerns	No concerns	Major concerns	Very low
PAR vs PLA	2	Some concerns	Some concerns	No concerns	No concerns	No concerns	Major concerns	Very low
REB vs PLA	1	Some concerns	Some concerns	No concerns	No concerns	Major concerns	Major concerns	Very low
SER vs PLA	2	Some concerns	Some concerns	No concerns	No concerns	No concerns	Major concerns	Very low
TIA vs PLA	1	Some concerns	Some concerns	No concerns	No concerns	No concerns	Major concerns	Very low
VEN vs PLA	3	Some concerns	Some concerns	No concerns	No concerns	No concerns	Major concerns	Very low
VIL vs PLA	1	No concerns	Some concerns	No concerns	Major concerns	No concerns	Major concerns	Very low
VOR vs PLA	2	No concerns	Some concerns	No concerns	No concerns	No concerns	Major concerns	Low
AGO vs AMI	0	Some concerns	Some concerns	No concerns	No concerns	No concerns	Major concerns	Very low
AGO vs BUP	0	Some concerns	Some concerns	No concerns	Major concern	No concerns	Major concerns	Very low
AGO vs CIT	0	Some concerns	Some concerns	No concerns	No concerns	Major concerns	Major concerns	Very low
AGO vs DES	0	Some concerns	Some concerns	No concerns	No concerns	No concerns	Major concerns	Very low

AGO vs DUL	0	Some concerns	Some concerns	No concerns	No concerns	No concerns	Major concerns	Very low
AGO vs ESC	0	Some concerns	Some concerns	No concerns	Major concern	No concerns	Major concerns	Very low
AGO vs FLUO	0	Some concerns	Some concerns	No concerns	Major concern	No concerns	Major concerns	Very low
AGO vs FLUV	0	Some concerns	Some concerns	No concerns	No concerns	No concerns	Major concerns	Very low
AGO vs LEV	0	Some concerns	Some concerns	No concerns	Major concern	No concerns	Major concerns	Very low
AGO vs MIL	0	Some concerns	Some concerns	No concerns	Major concern	No concerns	Major concerns	Very low
AGO vs MIR	0	Some concerns	Some concerns	No concerns	No concerns	No concerns	Major concerns	Very low
AGO vs NEF	0	Some concerns	Some concerns	No concerns	No concerns	No concerns	Major concerns	Very low
AGO vs PAR	0	Some concerns	Some concerns	No concerns	No concerns	No concerns	Major concerns	Very low
AGO vs REB	0	Some concerns	Some concerns	No concerns	No concerns	No concerns	Major concerns	Very low
AGO vs SER	0	Some concerns	Some concerns	No concerns	No concerns	No concerns	Major concerns	Very low
AGO vs TIA	0	Some concerns	Some concerns	No concerns	No concerns	No concerns	Major concerns	Very low
AGO vs VEN	0	Some concerns	Some concerns	No concerns	Major concern	No concerns	Major concerns	Very low
AGO vs VIL	0	Some concerns	Some concerns	No concerns	No concerns	No concerns	Major concerns	Very low
AGO vs VOR	0	Some concerns	Some concerns	No concerns	No concerns	No concerns	Major concerns	Very low
AMI vs BUP	0	Some concerns	Some concerns	No concerns	No concerns	No concerns	Major concerns	Very low
AMI vs CIT	0	Some concerns	Some concerns	No concerns	Major concern	No concerns	Major concerns	Very low
AMI vs DES	0	Some concerns	Some concerns	No concerns	No concerns	No concerns	Major concerns	Very low
AMI vs DUL	0	Some concerns	Some concerns	No concerns	Major concern	No concerns	Major concerns	Very low
AMI vs ESC	0	Some concerns	Some concerns	No concerns	No concerns	No concerns	Major concerns	Very low
AMI vs FLUO	0	Some concerns	Some concerns	No concerns	No concerns	No concerns	Major concerns	Very low
AMI vs FLUV	0	Some concerns	Some concerns	No concerns	No concerns	No concerns	Major concerns	Very low
AMI vs LEV	0	Some concerns	Some concerns	No concerns	No concerns	No concerns	Major concerns	Very low
AMI vs MIL	0	Some concerns	Some concerns	No concerns	No concerns	No concerns	Major concerns	Very low
AMI vs MIR	0	Some concerns	Some concerns	No concerns	Major concern	No concerns	Major concerns	Very low

AMI vs NEF	0	Some concerns	Some concerns	No concerns	No concerns	No concerns	Major concerns	Very low
AMI vs PAR	0	Some concerns	Some concerns	No concerns	Major concern	No concerns	Major concerns	Very low
AMI vs REB	0	Some concerns	Some concerns	No concerns	No concerns	No concerns	Major concerns	Very low
AMI vs SER	0	Some concerns	Some concerns	No concerns	No concerns	No concerns	Major concerns	Very low
AMI vs TIA	0	Some concerns	Some concerns	No concerns	No concerns	No concerns	Major concerns	Very low
AMI vs VEN	0	Some concerns	Some concerns	No concerns	No concerns	No concerns	Major concerns	Very low
AMI vs VIL	0	Some concerns	Some concerns	No concerns	No concerns	No concerns	Major concerns	Very low
AMI vs VOR	0	Some concerns	Some concerns	No concerns	No concerns	No concerns	Major concerns	Very low
BUP vs CIT	0	Some concerns	Some concerns	No concerns	No concerns	Major concerns	Major concerns	Very low
BUP vs DES	0	Some concerns	Some concerns	No concerns	No concerns	No concerns	Major concerns	Very low
BUP vs DUL	0	Some concerns	Some concerns	No concerns	No concerns	No concerns	Major concerns	Very low
BUP vs ESC	0	Some concerns	Some concerns	No concerns	Major concern	No concerns	Major concerns	Very low
BUP vs FLUO	0	Some concerns	Some concerns	No concerns	Major concern	No concerns	Major concerns	Very low
BUP vs FLUV	0	Some concerns	Some concerns	No concerns	No concerns	No concerns	Major concerns	Very low
BUP vs LEV	0	Some concerns	Some concerns	No concerns	Major concern	No concerns	Major concerns	Very low
BUP vs MIL	0	Some concerns	Some concerns	No concerns	Major concern	No concerns	Major concerns	Very low
BUP vs MIR	0	Some concerns	Some concerns	No concerns	No concerns	No concerns	Major concerns	Very low
BUP vs NEF	0	Some concerns	Some concerns	No concerns	No concerns	No concerns	Major concerns	Very low
BUP vs PAR	0	Some concerns	Some concerns	No concerns	No concerns	No concerns	Major concerns	Very low
BUP vs REB	0	Some concerns	Some concerns	No concerns	No concerns	No concerns	Major concerns	Very low
BUP vs SER	0	Some concerns	Some concerns	No concerns	No concerns	No concerns	Major concerns	Very low
BUP vs TIA	0	Some concerns	Some concerns	No concerns	No concerns	No concerns	Major concerns	Very low
BUP vs VEN	0	Some concerns	Some concerns	No concerns	No concerns	No concerns	Major concerns	Very low
BUP vs VIL	0	Some concerns	Some concerns	No concerns	No concerns	No concerns	Major concerns	Very low
BUP vs VOR	0	Some concerns	Some concerns	No concerns	No concerns	No concerns	Major concerns	Very low

CIT vs DES	0	Some concerns	Some concerns	No concerns	No concerns	No concerns	Major concerns	Very low
CIT vs DUL	0	Some concerns	Some concerns	No concerns	Major concern	No concerns	Major concerns	Very low
CIT vs ESC	0	Some concerns	Some concerns	No concerns	No concerns	No concerns	Major concerns	Very low
CIT vs FLUO	0	Some concerns	Some concerns	No concerns	No concerns	No concerns	Major concerns	Very low
CIT vs FLUV	0	Some concerns	Some concerns	No concerns	No concerns	No concerns	Major concerns	Very low
CIT vs LEV	0	Some concerns	Some concerns	No concerns	No concerns	No concerns	Major concerns	Very low
CIT vs MIL	0	Some concerns	Some concerns	No concerns	No concerns	No concerns	Major concerns	Very low
CIT vs MIR	0	Some concerns	Some concerns	No concerns	Major concern	No concerns	Major concerns	Very low
CIT vs NEF	0	Some concerns	Some concerns	No concerns	No concerns	No concerns	Major concerns	Very low
CIT vs PAR	0	Some concerns	Some concerns	No concerns	Major concern	No concerns	Major concerns	Very low
CIT vs REB	0	Some concerns	Some concerns	No concerns	No concerns	No concerns	Major concerns	Very low
CIT vs SER	0	Some concerns	Some concerns	No concerns	No concerns	Major concerns	Major concerns	Very low
CIT vs TIA	0	Some concerns	Some concerns	No concerns	No concerns	No concerns	Major concerns	Very low
CIT vs VEN	0	Some concerns	Some concerns	No concerns	No concerns	No concerns	Major concerns	Very low
CIT vs VIL	0	Some concerns	Some concerns	No concerns	No concerns	No concerns	Major concerns	Very low
CIT vs VOR	0	Some concerns	Some concerns	No concerns	No concerns	No concerns	Major concerns	Very low
DES vs DUL	0	Some concerns	Some concerns	No concerns	Major concern	No concerns	Major concerns	Very low
DES vs ESC	0	Some concerns	Some concerns	No concerns	Major concern	No concerns	Major concerns	Very low
DES vs FLUO	0	Some concerns	Some concerns	No concerns	Major concern	No concerns	Major concerns	Very low
DES vs FLUV	0	Some concerns	Some concerns	No concerns	No concerns	No concerns	Major concerns	Very low
DES vs LEV	0	Some concerns	Some concerns	No concerns	Major concern	No concerns	Major concerns	Very low
DES vs MIL	0	Some concerns	Some concerns	No concerns	No concerns	No concerns	Major concerns	Very low
DES vs MIR	0	Some concerns	Some concerns	No concerns	No concerns	No concerns	Major concerns	Very low
DES vs NEF	0	Some concerns	Some concerns	No concerns	No concerns	No concerns	Major concerns	Very low
DES vs PAR	0	Some concerns	Some concerns	No concerns	No concerns	No concerns	Major concerns	Very low

DES vs REB	0	Some concerns	Some concerns	No concerns	Major concern	No concerns	Major concerns	Very low
DES vs SER	0	Some concerns	Some concerns	No concerns	No concerns	No concerns	Major concerns	Very low
DES vs TIA	0	Some concerns	Some concerns	No concerns	No concerns	No concerns	Major concerns	Very low
DES vs VEN	0	Some concerns	Some concerns	No concerns	Major concern	No concerns	Major concerns	Very low
DES vs VIL	0	Some concerns	Some concerns	No concerns	No concerns	No concerns	Major concerns	Very low
DES vs VOR	0	Some concerns	Some concerns	No concerns	No concerns	No concerns	Major concerns	Very low
DUL vs ESC	0	Some concerns	Some concerns	No concerns	No concerns	No concerns	Major concerns	Very low
DUL vs FLUO	0	Some concerns	Some concerns	No concerns	No concerns	No concerns	Major concerns	Very low
DUL vs FLUV	0	Some concerns	Some concerns	No concerns	No concerns	No concerns	Major concerns	Very low
DUL vs LEV	0	Some concerns	Some concerns	No concerns	No concerns	No concerns	Major concerns	Very low
DUL vs MIL	0	Some concerns	Some concerns	No concerns	No concerns	No concerns	Major concerns	Very low
DUL vs MIR	0	Some concerns	Some concerns	No concerns	Major concern	No concerns	Major concerns	Very low
DUL vs NEF	0	Some concerns	Some concerns	No concerns	No concerns	No concerns	Major concerns	Very low
DUL vs PAR	0	Some concerns	Some concerns	No concerns	Major concern	No concerns	Major concerns	Very low
DUL vs REB	0	Some concerns	Some concerns	No concerns	Major concern	No concerns	Major concerns	Very low
DUL vs SER	0	Some concerns	Some concerns	No concerns	No concerns	No concerns	Major concerns	Very low
DUL vs TIA	0	Some concerns	Some concerns	No concerns	No concerns	No concerns	Major concerns	Very low
DUL vs VEN	0	Some concerns	Some concerns	No concerns	Major concern	No concerns	Major concerns	Very low
DUL vs VIL	0	Some concerns	Some concerns	No concerns	No concerns	Major concerns	Major concerns	Very low
DUL vs VOR	0	Some concerns	Some concerns	No concerns	Major concern	No concerns	Major concerns	Very low
ESC vs FLUO	0	Some concerns	Some concerns	No concerns	Major concern	No concerns	Major concerns	Very low
ESC vs FLUV	0	Some concerns	Some concerns	No concerns	No concerns	No concerns	Major concerns	Very low
ESC vs LEV	0	Some concerns	Some concerns	No concerns	Major concern	No concerns	Major concerns	Very low
ESC vs MIL	0	Some concerns	Some concerns	No concerns	Major concern	No concerns	Major concerns	Very low
ESC vs MIR	0	Some concerns	Some concerns	No concerns	No concerns	No concerns	Major concerns	Very low

ESC vs NEF	0	Some concerns	Some concerns	No concerns	No concerns	No concerns	Major concerns	Very low
ESC vs PAR	0	Some concerns	Some concerns	No concerns	No concerns	No concerns	Major concerns	Very low
ESC vs REB	0	Some concerns	Some concerns	No concerns	Major concern	No concerns	Major concerns	Very low
ESC vs SER	0	Some concerns	Some concerns	No concerns	No concerns	No concerns	Major concerns	Very low
ESC vs TIA	0	Some concerns	Some concerns	No concerns	No concerns	No concerns	Major concerns	Very low
ESC vs VEN	0	Some concerns	Some concerns	No concerns	Major concern	No concerns	Major concerns	Very low
ESC vs VIL	0	Some concerns	Some concerns	No concerns	No concerns	No concerns	Major concerns	Very low
ESC vs VOR	0	Some concerns	Some concerns	No concerns	Major concern	No concerns	Major concerns	Very low
FLUO vs FLUV	0	Some concerns	Some concerns	No concerns	No concerns	No concerns	Major concerns	Very low
FLUO vs LEV	0	Some concerns	Some concerns	No concerns	Major concern	No concerns	Major concerns	Very low
FLUO vs MIL	0	Some concerns	Some concerns	No concerns	Major concern	No concerns	Major concerns	Very low
FLUO vs MIR	0	Some concerns	Some concerns	No concerns	No concerns	No concerns	Major concerns	Very low
FLUO vs NEF	0	Some concerns	Some concerns	No concerns	No concerns	No concerns	Major concerns	Very low
FLUO vs PAR	0	Some concerns	Some concerns	No concerns	No concerns	No concerns	Major concerns	Very low
FLUO vs REB	0	Some concerns	Some concerns	No concerns	Major concern	No concerns	Major concerns	Very low
FLUO vs SER	0	Some concerns	Some concerns	No concerns	No concerns	No concerns	Major concerns	Very low
FLUO vs TIA	0	Some concerns	Some concerns	No concerns	No concerns	No concerns	Major concerns	Very low
FLUO vs VEN	0	Some concerns	Some concerns	No concerns	Major concern	No concerns	Major concerns	Very low
FLUO vs VIL	0	Some concerns	Some concerns	No concerns	No concerns	No concerns	Major concerns	Very low
FLUO vs VOR	0	Some concerns	Some concerns	No concerns	Major concern	No concerns	Major concerns	Very low
FLUV vs LEV	0	Some concerns	Some concerns	No concerns	No concerns	No concerns	Major concerns	Very low
FLUV vs MIL	0	Some concerns	Some concerns	No concerns	No concerns	No concerns	Major concerns	Very low
FLUV vs MIR	0	Some concerns	Some concerns	No concerns	No concerns	No concerns	Major concerns	Very low
FLUV vs NEF	0	Some concerns	Some concerns	No concerns	No concerns	No concerns	Major concerns	Very low
FLUV vs PAR	0	Some concerns	Some concerns	No concerns	No concerns	No concerns	Major concerns	Very low

FLUV vs REB	0	Some concerns	Some concerns	No concerns	No concerns	No concerns	Major concerns	Very low
FLUV vs SER	0	Some concerns	Some concerns	No concerns	No concerns	No concerns	Major concerns	Very low
FLUV vs TIA	0	Some concerns	Some concerns	No concerns	Major concern	No concerns	Major concerns	Very low
FLUV vs VEN	0	Some concerns	Some concerns	No concerns	No concerns	No concerns	Major concerns	Very low
FLUV vs VIL	0	Some concerns	Some concerns	No concerns	No concerns	Major concerns	Major concerns	Very low
FLUV vs VOR	0	Some concerns	Some concerns	No concerns	No concerns	No concerns	Major concerns	Very low
LEV vs MIL	0	Some concerns	Some concerns	No concerns	Major concern	No concerns	Major concerns	Very low
LEV vs MIR	0	Some concerns	Some concerns	No concerns	No concerns	No concerns	Major concerns	Very low
LEV vs NEF	0	Some concerns	Some concerns	No concerns	No concerns	No concerns	Major concerns	Very low
LEV vs PAR	0	Some concerns	Some concerns	No concerns	No concerns	No concerns	Major concerns	Very low
LEV vs REB	0	Some concerns	Some concerns	No concerns	Major concern	No concerns	Major concerns	Very low
LEV vs SER	0	Some concerns	Some concerns	No concerns	No concerns	No concerns	Major concerns	Very low
LEV vs TIA	0	Some concerns	Some concerns	No concerns	No concerns	No concerns	Major concerns	Very low
LEV vs VEN	0	Some concerns	Some concerns	No concerns	Major concern	No concerns	Major concerns	Very low
LEV vs VIL	0	No concerns	Some concerns	No concerns	No concerns	No concerns	Major concerns	Very low
LEV vs VOR	0	No concerns	Some concerns	No concerns	Major concern	No concerns	Major concerns	Very low
MIL vs MIR	0	Some concerns	Some concerns	No concerns	No concerns	No concerns	Major concerns	Very low
MIL vs NEF	0	Some concerns	Some concerns	No concerns	No concerns	No concerns	Major concerns	Very low
MIL vs PAR	0	Some concerns	Some concerns	No concerns	No concerns	No concerns	Major concerns	Very low
MIL vs REB	0	Some concerns	Some concerns	No concerns	No concerns	No concerns	Major concerns	Very low
MIL vs SER	0	Some concerns	Some concerns	No concerns	No concerns	No concerns	Major concerns	Very low
MIL vs TIA	0	Some concerns	Some concerns	No concerns	No concerns	No concerns	Major concerns	Very low
MIL vs VEN	0	Some concerns	Some concerns	No concerns	No concerns	No concerns	Major concerns	Very low
MIL vs VIL	0	Some concerns	Some concerns	No concerns	No concerns	No concerns	Major concerns	Very low
MIL vs VOR	0	Some concerns	Some concerns	No concerns	No concerns	No concerns	Major concerns	Very low

MIR vs NEF	0	Some concerns	Some concerns	No concerns	No concerns	No concerns	Major concerns	Very low
MIR vs PAR	0	Some concerns	Some concerns	No concerns	Major concerns	No concerns	Major concerns	Very low
MIR vs REB	0	Some concerns	Some concerns	No concerns	No concerns	No concerns	Major concerns	Very low
MIR vs SER	0	Some concerns	Some concerns	No concerns	Major concerns	Major concerns	Major concerns	Very low
MIR vs TIA	0	Some concerns	Some concerns	No concerns	No concerns	No concerns	Major concerns	Very low
MIR vs VEN	0	Some concerns	Some concerns	No concerns	No concerns	No concerns	Major concerns	Very low
MIR vs VIL	0	Some concerns	Some concerns	No concerns	No concerns	Major concerns	Major concerns	Very low
MIR vs VOR	0	Some concerns	Some concerns	No concerns	No concerns	No concerns	Major concerns	Very low
NEF vs PAR	0	Some concerns	Some concerns	No concerns	No concerns	No concerns	Major concerns	Very low
NEF vs REB	0	Some concerns	Some concerns	No concerns	No concerns	No concerns	Major concerns	Very low
NEF vs SER	0	Some concerns	Some concerns	No concerns	Major concerns	No concerns	Major concerns	Very low
NEF vs TIA	0	Some concerns	Some concerns	No concerns	No concerns	No concerns	Major concerns	Very low
NEF vs VEN	0	Some concerns	Some concerns	No concerns	No concerns	No concerns	Major concerns	Very low
NEF vs VIL	0	Some concerns	Some concerns	No concerns	No concerns	Major concerns	Major concerns	Very low
NEF vs VOR	0	Some concerns	Some concerns	No concerns	No concerns	No concerns	Major concerns	Very low
PAR vs REB	0	Some concerns	Some concerns	No concerns	No concerns	No concerns	Major concerns	Very low
PAR vs SER	0	Some concerns	Some concerns	No concerns	No concerns	Major concerns	Major concerns	Very low
PAR vs TIA	0	Some concerns	Some concerns	No concerns	No concerns	No concerns	Major concerns	Very low
PAR vs VEN	0	Some concerns	Some concerns	No concerns	No concerns	No concerns	Major concerns	Very low
PAR vs VIL	0	Some concerns	Some concerns	No concerns	No concerns	No concerns	Major concerns	Very low
PAR vs VOR	0	Some concerns	Some concerns	No concerns	No concerns	No concerns	Major concerns	Very low
REB vs SER	0	Some concerns	Some concerns	No concerns	No concerns	No concerns	Major concerns	Very low
REB vs TIA	0	Some concerns	Some concerns	No concerns	No concerns	No concerns	Major concerns	Very low
REB vs VEN	0	Some concerns	Some concerns	No concerns	Major concerns	No concerns	Major concerns	Very low
REB vs VIL	0	Some concerns	Some concerns	No concerns	No concerns	No concerns	Major concerns	Very low

REB vs VOR	0	Some concerns	Some concerns	No concerns	Major concerns	No concerns	Major concerns	Very low
SER vs TIA	0	Some concerns	Some concerns	No concerns	No concerns	No concerns	Major concerns	Very low
SER vs VEN	0	Some concerns	Some concerns	No concerns	No concerns	No concerns	Major concerns	Very low
SER vs VIL	0	Some concerns	Some concerns	No concerns	No concerns	No concerns	Major concerns	Very low
SER vs VOR	0	Some concerns	Some concerns	No concerns	No concerns	No concerns	Major concerns	Very low
TIA vs VEN	0	Some concerns	Some concerns	No concerns	No concerns	No concerns	Major concerns	Very low
TIA vs VIL	0	Some concerns	Some concerns	No concerns	No concerns	Major concerns	Major concerns	Very low
TIA vs VOR	0	Some concerns	Some concerns	No concerns	No concerns	No concerns	Major concerns	Very low
VEN vs VIL	0	Some concerns	Some concerns	No concerns	No concerns	No concerns	Major concerns	Very low
VEN vs VOR	0	Some concerns	Some concerns	No concerns	Major concerns	No concerns	Major concerns	Very low
VIL vs VOR	0	No concerns	Some concerns	No concerns	No concerns	No concerns	Major concerns	Very low

Appendix S2. All-cause discontinuation (K = 28, n = 8317).

League table (risk ratio with 95% credible interval)

PLA																
0.778 (0.542, 1.111)	AGO															
0.787 (0.500, 1.238)	1.010 (0.572, 1.815)	BUP														
**0.589 (0.417, 0.834)**	0.758 (0.464, 1.250)	0.748 (0.429, 1.331)	DES													
0.784 (0.544, 1.156)	1.010 (0.604, 1.718)	1.003 (0.566, 1.814)	1.336 (0.805, 2.246)	DUL												
0.742 (0.467, 1.159)	0.954 (0.527, 1.706)	0.944 (0.489, 1.791)	1.257 (0.706, 2.204)	0.950 (0.513, 1.670)	ESC											
0.863 (0.657, 1.137)	1.114 (0.711, 1.754)	1.098 (0.654, 1.856)	1.468 (0.936, 2.271)	1.096 (0.691, 1.752)	1.165 (0.692, 1.984)	FLUO										
1.207 (0.774, 1.852)	1.548 (0.886, 2.713)	1.530 (0.806, 2.892)	**2.040 (1.166, 3.523)**	1.530 (0.847, 2.711)	1.628 (0.866, 3.045)	1.398 (0.824, 2.343)	LEV									
0.726 (0.430, 1.199)	0.934 (0.495, 1.766)	0.918 (0.463, 1.851)	1.233 (0.657, 2.278)	0.919 (0.480, 1.733)	0.976 (0.490, 1.925)	0.838 (0.466, 1.476)	0.603 (0.304, 1.194)	MIL								
0.857 (0.490, 1.504)	1.102 (0.560, 2.128)	1.093 (0.529, 2.243)	1.455 (0.746, 2.806)	1.089 (0.545, 2.085)	1.155 (0.572, 2.428)	0.996 (0.533, 1.820)	0.711 (0.351, 1.455)	1.185 (0.558, 2.533)	NEF							
**0.523 (0.327, 0.817)**	0.674 (0.379, 1.178)	0.667 (0.348, 1.251)	0.886 (0.495, 1.548)	0.666 (0.364, 1.171)	0.706 (0.374, 1.348)	0.603 (0.353, 1.008)	**0.432 (0.230, 0.820)**	0.723 (0.357, 1.448)	0.608 (0.301, 1.246)	PAR						
0.843 (0.526, 1.360)	1.085 (0.600, 1.956)	1.074 (0.559, 2.053)	1.424 (0.801, 2.599)	1.070 (0.584, 1.948)	1.139 (0.594, 2.223)	0.974 (0.565, 1.698)	0.701 (0.370, 1.327)	1.160 (0.587, 2.392)	0.982 (0.471, 2.097)	1.614 (0.844, 3.167)	REB					

**0.681 (0.492, 0.961)**	0.875 (0.548, 1.445)	0.865 (0.504, 1.523)	1.152 (0.719, 1.891)	0.865 (0.528, 1.442)	0.918 (0.536, 1.625)	0.786 (0.515, 1.214)	**0.566 (0.332, 0.990)**	0.939 (0.514, 1.792)	0.795 (0.419, 1.564)	1.307 (0.758, 2.318)	0.807 (0.458, 1.435)	SER				
0.667 (0.407, 1.102)	0.862 (0.466, 1.554)	0.848 (0.444, 1.674)	1.132 (0.631, 2.083)	0.851 (0.453, 1.584)	0.899 (0.462, 1.743)	0.773 (0.436, 1.363)	0.555 (0.287, 1.081)	0.924 (0.449, 1.883)	0.778 (0.375, 1.661)	1.269 (0.665, 2.479)	0.793 (0.391, 1.529)	0.983 (0.542, 1.752)	TIA			
**0.681 (0.522, 0.883)**	0.874 (0.560, 1.370)	0.868 (0.507, 1.464)	1.157 (0.752, 1.798)	0.866 (0.547, 1.351)	0.921 (0.548, 1.549)	0.788 (0.535, 1.153)	**0.567 (0.338, 0.960)**	0.939 (0.526, 1.694)	0.795 (0.428, 1.504)	1.304 (0.777, 2.217)	0.808 (0.470, 1.372)	1.002 (0.644, 1.511)	1.020 (0.583, 1.780)	VEN		
1.209 (0.744, 1.976)	1.558 (0.849, 2.872)	1.542 (0.785, 3.008)	**2.050 (1.132, 3.718)**	1.543 (0.819, 2.843)	1.628 (0.843, 3.240)	1.396 (0.811, 2.448)	1.010 (0.521, 2.012)	1.676 (0.808, 3.521)	1.412 (0.669, 2.970)	**2.317 (1.190, 4.572)**	1.436 (0.731, 2.854)	1.777 (0.986, 3.185)	1.813 (0.904, 3.618)	**1.774 (1.020, 3.112)**	VIL	
**0.768 (0.518, 0.998)**	0.922 (0.565, 1.504)	0.910 (0.528, 1.607)	1.215 (0.756, 1.962)	0.909 (0.558, 1.479)	0.968 (0.551, 1.705)	0.828 (0.543, 1.283)	0.594 (0.347, 1.043)	0.987 (0.542, 1.831)	0.838 (0.448, 1.612)	1.372 (0.794, 2.415)	0.850 (0.470, 1.503)	1.051 (0.658, 1.669)	1.071 (0.589, 1.927)	1.047 (0.690, 1.613)	0.591 (0.326, 1.078)	VOR

Evaluation of heterogeneity

Network meta-analysis

Between study variance (τ^2): 0.030

Heterogeneity assessment: Moderate

Pairwise meta-analysis

	Risk ratio (95% confidence interval)	I^2
AGO vs PLA	0.778 (0.583, 1.040)	75.5%
BUP vs PLA	0.783 (0.558, 1.101)	na
DES vs PLA	**0.592 (0.451, 0.777)**	0.0%
DUL vs PLA	0.787 (0.576, 1.073)	18.2%
ESC vs PLA	0.738 (0.519, 1.047)	na
FLUO vs PLA	0.868 (0.694, 1.084)	80.8%
LEV vs PLA	1.198 (0.806, 1.779)	0.0%

MIL vs PLA	0.725 (0.472, 1.112)	na
NEF vs PLA	0.857 (0.533, 1.377)	na
PAR vs PLA	**0.520 (0.346, 0.780)**	0.0%
REB vs PLA	0.844 (0.585, 1.220)	na
SER vs PLA	**0.684 (0.528, 0.885)**	37.7%
TIA vs PLA	**0.667 (0.450, 0.987)**	na
VEN vs PLA	**0.682 (0.559, 0.832)**	0.0%
VIL vs PLA	1.207 (0.822, 1.774)	na
VOR vs PLA	**0.771 (0.601, 0.988)**	28.7%

Forest plot (vs placebo, the numbers are risk ratios with 95% credible interval)

Treatments are ranked according to their SCURA.

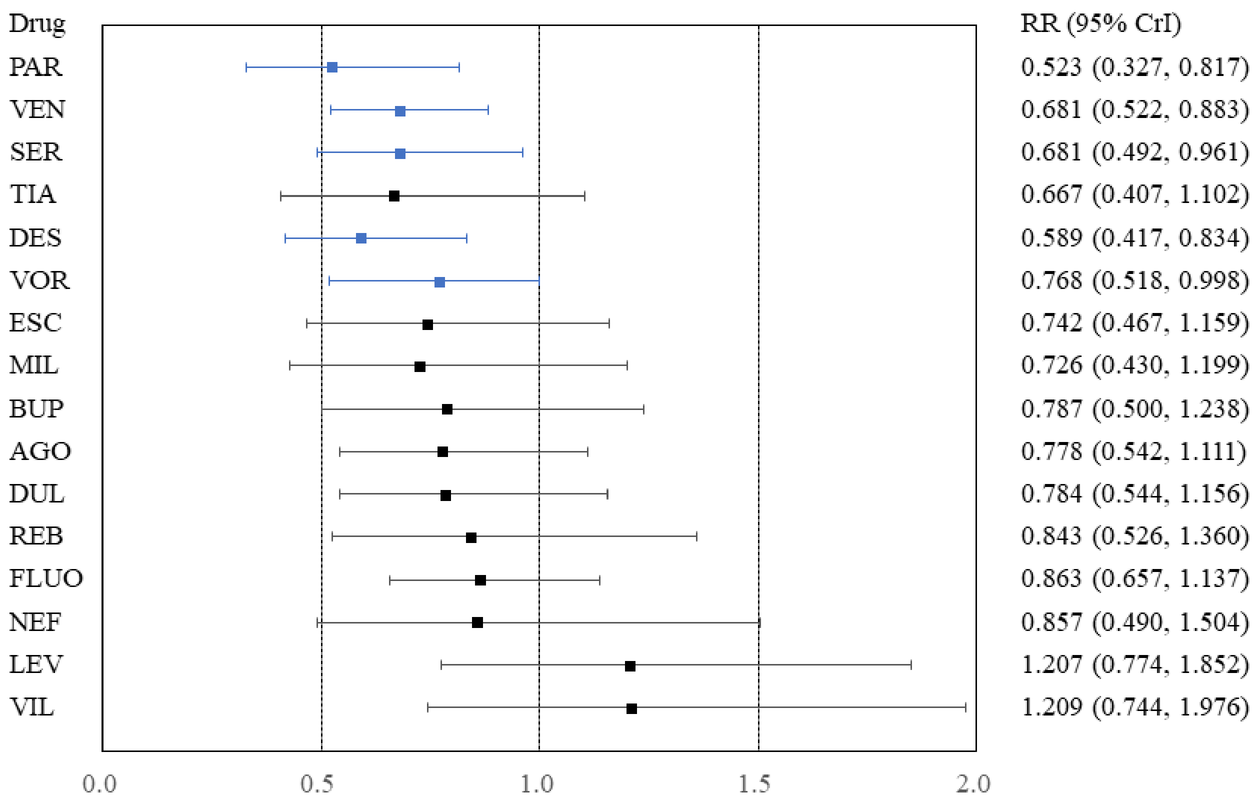

Appendix S3. Discontinuation due to adverse events (K = 27, n = 8152).

League table (risk ratio with 95% credible interval)

PLA															
1.247 (0.272, 6.608)	AGO														
4.364 (0.780, 35.958)	3.652 (0.340, 43.466)	BUP													
0.528 (0.186, 1.199)	0.416 (0.059, 2.320)	**0.119 (0.011, 0.775)**	DES												
1.427 (0.443, 4.875)	1.155 (0.144, 8.167)	0.325 (0.028, 2.576)	2.725 (0.705, 14.017)	DUL											
0.530 (0.114, 2.163)	0.423 (0.047, 3.277)	0.119 (0.009, 1.109)	1.013 (0.183, 5.803)	0.363 (0.052, 2.231)	ESC										
2.296 (0.402, 20.441)	1.839 (0.167, 27.670)	0.523 (0.033, 7.640)	4.514 (0.679, 48.343)	1.614 (0.200, 18.069)	4.567 (0.480, 57.855)	FLUO									
1.789 (0.557, 6.891)	1.439 (0.196, 10.820)	0.390 (0.037, 3.700)	3.441 (0.817, 18.673)	1.243 (0.223, 7.690)	3.427 (0.553, 25.164)	0.795 (0.069, 6.931)	LEV								
5.538 (0.966, 48.933)	4.564 (0.372, 59.035)	1.266 (0.081, 19.481)	**10.796 (1.563, 122.996)**	3.901 (0.494, 45.122)	**10.756 (1.157, 150.367)**	2.441 (0.156, 42.772)	3.166 (0.339, 34.040)	MIR							
0.230 (0.005, 2.805)	0.174 (0.003, 3.659)	0.050 (0.001, 1.090)	0.442 (0.009, 6.788)	0.161 (0.003, 2.549)	0.436 (0.008, 8.837)	0.097 (0.001, 2.307)	0.125 (0.002, 2.043)	**0.038 (0.001, 0.856)**	NEF						
1.790 (0.303, 11.628)	1.482 (0.118, 14.897)	0.387 (0.026, 4.982)	3.491 (0.498, 28.246)	1.263 (0.137, 11.375)	3.403 (0.348, 38.781)	0.772 (0.044, 9.976)	0.985 (0.104, 8.417)	0.317 (0.019, 3.973)	8.074 (0.353, 419.030)	PAR					
3.343 (0.530, 30.667)	2.664 (0.231, 37.525)	0.743 (0.047, 13.040)	6.491 (0.886, 70.662)	2.281 (0.265, 27.275)	6.491 (0.654, 89.395)	1.417 (0.095, 22.723)	1.871 (0.190, 22.618)	0.598 (0.034, 9.674)	15.563 (0.626, 1, 026.783)	1.836 (0.137, 32.895)	REB				

**4.447 (1.161, 23.812)**	3.682 (0.427, 32.837)	0.986 (0.085, 11.267)	**8.636 (1.751, 61.770)**	3.085 (0.528, 24.134)	**8.513 (1.327, 78.867)**	1.968 (0.159, 21.842)	2.547 (0.366, 18.785)	0.799 (0.067, 9.376)	**20.141 (1.110, 172.628)**	2.508 (0.267, 27.907)	1.383 (0.108, 15.954)	SER			
0.583 (0.260, 1.268)	0.462 (0.073, 2.481)	**0.131 (0.014, 0.880)**	1.102 (0.361, 4.115)	0.411 (0.093, 1.636)	1.107 (0.218, 6.270)	0.250 (0.026, 1.695)	0.330 (0.068, 1.273)	**0.105 (0.011, 0.664)**	2.563 (0.185, 119.048)	0.319 (0.044, 2.239)	0.172 (0.016, 1.276)	**0.129 (0.021, 0.602)**	VEN		
1.444 (0.230, 13.077)	1.160 (0.092, 17.200)	0.317 (0.020, 5.298)	2.808 (0.369, 33.237)	1.005 (0.107, 11.441)	2.786 (0.265, 37.414)	0.629 (0.037, 10.429)	0.811 (0.080, 9.209)	0.251 (0.014, 4.592)	6.432 (0.260, 468.132)	0.806 (0.059, 13.202)	0.424 (0.026, 7.249)	0.318 (0.026, 4.626)	2.463 (0.336, 26.329)	VIL	
2.096 (0.767, 6.092)	1.692 (0.237, 10.607)	0.481 (0.046, 3.783)	**4.039 (1.116, 18.330)**	1.471 (0.315, 7.021)	4.049 (0.739, 24.892)	0.910 (0.087, 6.722)	1.199 (0.211, 5.799)	0.370 (0.034, 2.847)	9.391 (0.651, 493.497)	1.195 (0.144, 10.160)	0.630 (0.057, 5.177)	0.469 (0.070, 2.737)	**3.625 (1.001, 14.211)**	1.479 (0.123, 12.926)	VOR

Evaluation of heterogeneity

Network meta-analysis

Between study variance (τ^2): 0.102

Heterogeneity assessment: Moderate

Pairwise meta-analysis

	Risk ratio (95% confidence interval)	I^2
AGO vs PLA	1.214 (0.253, 5.818)	61.4%
BUP vs PLA	4.057 (0.872, 18.882)	na
DES vs PLA	**0.576 (0.355, 0.934)**	0.0%
DUL vs PLA	1.375 (0.554, 3.412)	0.0%
ESC vs PLA	0.514 (0.186, 1.421)	na
FLUO vs PLA	1.931 (0.438, 8.510)	na
LEV vs PLA	1.643 (0.593, 4.551)	0.0%
MIR vs PLA	**4.737 (1.057, 21.221)**	na
NEF vs PLA	0.338 (0.036, 3.170)	na

PAR vs PLA	1.642 (0.409, 6.600)	na
REB vs PLA	2.917 (0.599, 14.212)	na
SER vs PLA	**3.504 (1.015, 12.104)**	15.9%
VEN vs PLA	0.604 (0.358, 1.019)	0.0%
VIL vs PLA	1.290 (0.253, 6.589)	na
VOR vs PLA	2.020 (0.971, 4.203)	29.4%

Forest plot (vs placebo, the numbers are risk ratios with 95% credible interval)

Treatments are ranked according to their SCURA.

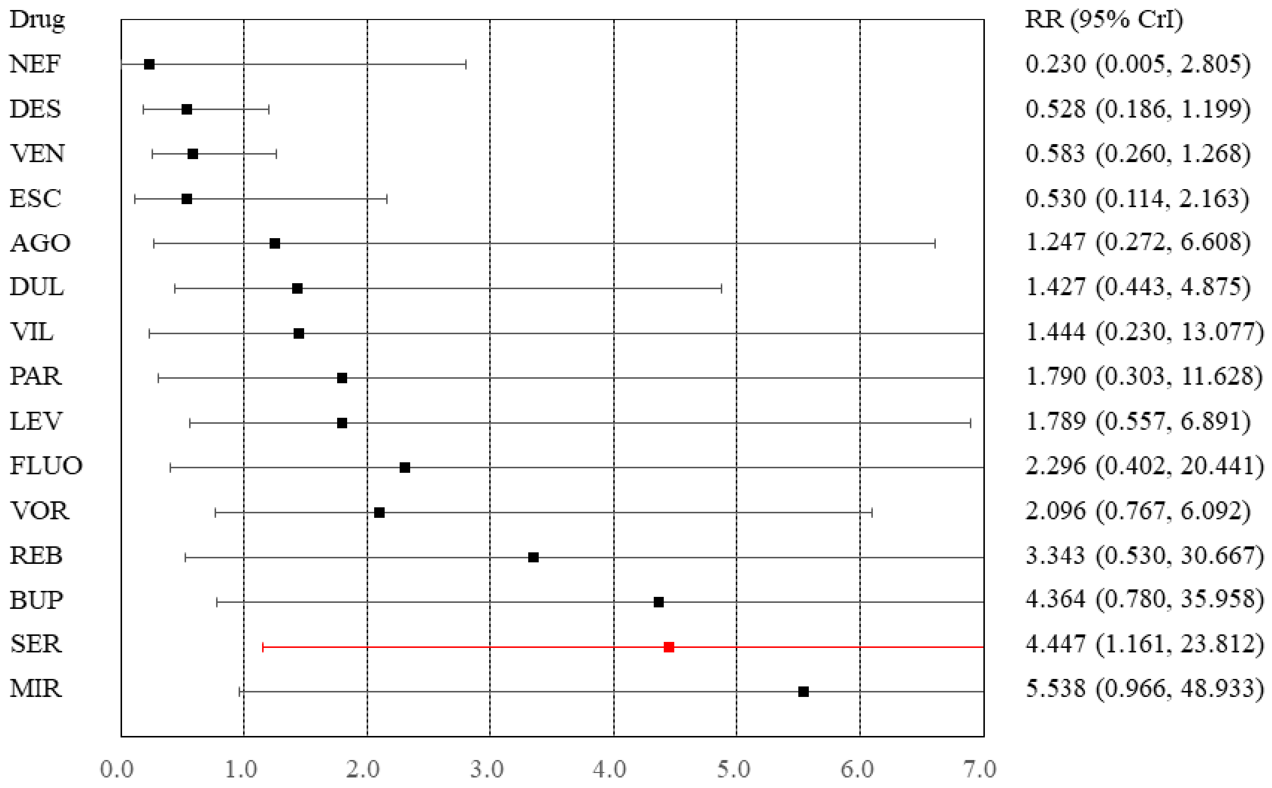

Appendix S4. Nausea/vomiting (K = 20, n = 6259).

League table (risk ratio with 95% credible interval)

PLA													
0.428 (0.042, 2.656)	BUP												
0.627 (0.248, 1.584)	1.480 (0.204, 17.965)	CIT											
**3.011 (1.121, 8.879)**	7.127 (0.860, 91.015)	**4.843 (1.243, 19.758)**	DES										
1.258 (0.469, 3.579)	3.016 (0.382, 36.949)	2.013 (0.523, 7.801)	0.421 (0.095, 1.802)	DUL									
1.297 (0.383, 5.715)	3.149 (0.326, 49.326)	2.086 (0.456, 11.515)	0.428 (0.085, 2.680)	1.047 (0.201, 5.920)	ESC								
0.840 (0.401, 1.830)	1.980 (0.291, 21.680)	1.336 (0.418, 4.481)	**0.279 (0.073, 0.990)**	0.668 (0.179, 2.317)	0.642 (0.124, 2.753)	FLUO							
0.368 (0.094, 1.258)	0.881 (0.089, 10.669)	0.591 (0.119, 2.680)	**0.121 (0.021, 0.566)**	0.293 (0.055, 1.395)	0.279 (0.039, 1.679)	0.445 (0.095, 1.804)	FLUV						
1.867 (0.792, 4.535)	4.389 (0.633, 51.116)	2.978 (0.826, 10.418)	0.617 (0.155, 2.344)	1.472 (0.375, 5.729)	1.425 (0.258, 6.498)	2.211 (0.691, 6.954)	**5.058 (1.138, 25.973)**	LEV					
1.740 (0.490, 6.794)	4.165 (0.460, 57.187)	2.740 (0.579, 13.408)	0.580 (0.115, 3.008)	1.372 (0.269, 7.074)	1.311 (0.191, 8.473)	2.077 (0.474, 9.551)	4.743 (0.842, 31.400)	0.923 (0.201, 4.611)	NEF				
**2.660 (1.015, 9.065)**	6.592 (0.811, 84.072)	**4.326 (1.110, 19.561)**	0.895 (0.216, 4.250)	2.135 (0.505, 9.982)	2.072 (0.342, 12.462)	3.202 (0.910, 13.394)	**7.477 (1.503, 44.197)**	1.426 (0.384, 6.502)	1.541 (0.305, 9.336)	SER			
1.239 (0.623, 2.436)	2.932 (0.424, 30.193)	1.972 (0.614, 6.315)	0.413 (0.115, 1.337)	0.982 (0.281, 3.159)	0.958 (0.178, 3.907)	1.465 (0.516, 4.079)	3.368 (0.806, 15.469)	0.659 (0.221, 2.000)	0.710 (0.154, 2.957)	0.460 (0.112, 1.556)	VEN		
1.134 (0.368, 3.545)	2.662 (0.308, 34.599)	1.785 (0.423, 7.965)	0.371 (0.084, 1.663)	0.892 (0.193, 4.078)	0.877 (0.133, 4.777)	1.346 (0.350, 5.138)	3.060 (0.600, 17.458)	0.607 (0.149, 2.518)	0.653 (0.116, 3.562)	0.424 (0.079, 1.912)	0.924 (0.248, 3.662)	VIL	
**3.222 (1.380, 9.254)**	**7.799 (1.066, 86.219)**	**5.201 (1.471, 20.750)**	1.080 (0.285, 4.570)	2.566 (0.687, 10.754)	2.484 (0.475, 13.441)	**3.890 (1.192, 14.464)**	**8.980 (2.005, 48.817)**	1.755 (0.505, 6.565)	1.911 (0.358, 9.589)	1.218 (0.263, 5.128)	2.611 (0.871, 9.288)	2.895 (0.715, 12.942)	VOR

Evaluation of heterogeneity

Network meta-analysis

Between study variance (τ^2): 0.040

Heterogeneity assessment: Moderate

Pairwise meta-analysis

	Risk ratio (95% confidence interval)	I^2
BUP vs PLA	0.493 (0.091, 2.663)	na

CIT vs PLA	0.630 (0.299, 1.328)	0.0%
DES vs PLA	**2.921 (1.412, 6.042)**	na
DUL vs PLA	1.264 (0.573, 2.790)	na
ESC vs PLA	1.285 (0.414, 3.985)	na
FLUO vs PLA	0.828 (0.468, 1.464)	0.0%
FLUV vs PLA	0.392 (0.141, 1.088)	na
LEV vs PLA	1.768 (0.868, 3.605)	0.0%
NEF vs PLA	1.625 (0.561, 4.706)	na
SER vs PLA	2.476 (0.985, 6.225)	0.0%
VEN vs PLA	1.248 (0.775, 2.008)	0.0%
VIL vs PLA	1.109 (0.460, 2.674)	na
VOR vs PLA	**3.088 (1.438, 6.630)**	0.0%

Forest plot (vs placebo, the numbers are risk ratios with 95% credible interval)

Treatments are ranked according to their SCURA.

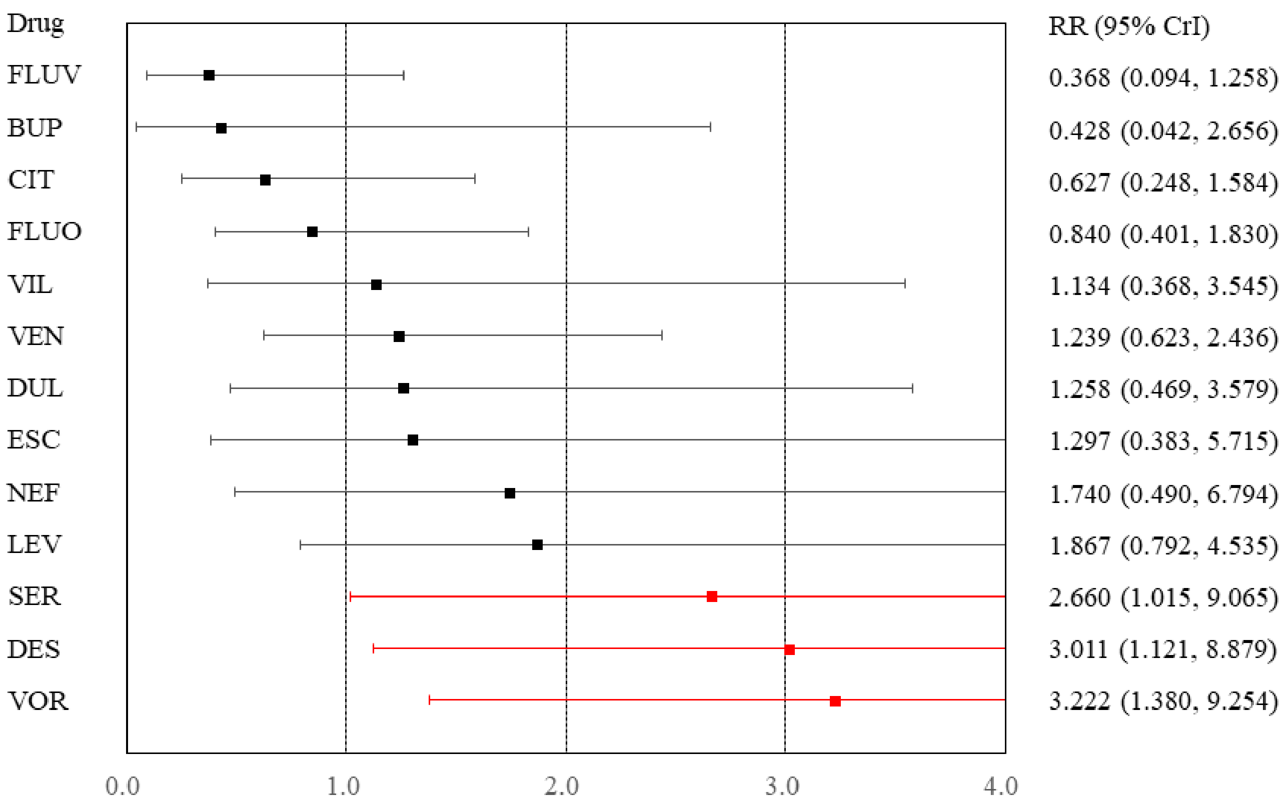

Appendix S5. Dizziness (K = 17, n = 5326).

League table (risk ratio with 95% credible interval)

PLA											
3.589 (0.342, 159.412)	BUP										
0.666 (0.287, 1.788)	0.190 (0.004, 2.406)	CIT									
0.437 (0.147, 1.211)	0.121 (0.003, 1.597)	0.652 (0.140, 2.332)	DES								
0.555 (0.153, 1.837)	0.147 (0.003, 2.290)	0.811 (0.160, 3.579)	1.255 (0.249, 6.446)	DUL							
0.818 (0.347, 1.898)	0.226 (0.004, 2.801)	1.243 (0.316, 3.961)	1.872 (0.493, 7.360)	1.499 (0.339, 6.833)	FLUO						
0.317 (0.084, 1.098)	0.089 (0.002, 1.259)	0.474 (0.092, 2.104)	0.737 (0.130, 3.662)	0.585 (0.092, 3.379)	0.389 (0.077, 1.680)	LEV					
1.068 (0.158, 6.064)	0.281 (0.005, 5.396)	1.572 (0.186, 10.523)	2.461 (0.280, 18.555)	1.948 (0.198, 16.664)	1.325 (0.163, 8.860)	3.322 (0.366, 31.447)	MIR				
0.799 (0.339, 1.923)	0.224 (0.005, 2.803)	1.188 (0.328, 3.940)	1.824 (0.502, 7.525)	1.474 (0.332, 6.767)	0.980 (0.313, 3.344)	2.518 (0.566, 12.434)	0.757 (0.112, 6.375)	SER			
**0.596 (0.379, 0.987)**	0.164 (0.004, 2.008)	0.885 (0.248, 2.607)	1.366 (0.384, 4.870)	1.081 (0.257, 5.379)	0.730 (0.240, 2.132)	1.850 (0.445, 8.733)	0.557 (0.084, 4.140)	0.741 (0.228, 2.250)	VEN		
0.339 (0.068, 1.592)	0.091 (0.002, 1.655)	0.501 (0.075, 2.856)	0.763 (0.115, 5.078)	0.615 (0.080, 4.192)	0.410 (0.066, 2.316)	1.055 (0.139, 8.194)	0.309 (0.029, 3.749)	0.418 (0.071, 2.378)	0.559 (0.099, 3.196)	VIL	
1.591 (0.314, 10.334)	0.434 (0.009, 9.337)	2.393 (0.341, 18.129)	3.684 (0.538, 32.448)	2.909 (0.380, 28.171)	1.938 (0.312, 15.171)	5.086 (0.679, 50.758)	1.472 (0.136, 20.874)	2.008 (0.299, 15.060)	2.704 (0.427, 19.685)	4.958 (0.503, 50.633)	VOR

Evaluation of heterogeneity

Network meta-analysis

Between study variance (τ^2): 0.066

Heterogeneity assessment: Moderate

Pairwise meta-analysis

	Risk ratio (95% confidence interval)	I^2
BUP vs PLA	2.958 (0.310, 28.208)	na
CIT vs PLA	0.589 (0.308, 1.129)	29.2%
DES vs PLA	**0.455 (0.242, 0.856)**	na

DUL vs PLA	0.567 (0.230, 1.400)	na
FLUO vs PLA	0.807 (0.434, 1.501)	7.4%
LEV vs PLA	0.340 (0.112, 1.037)	0.0%
MIR vs PLA	1.053 (0.219, 5.056)	na
SER vs PLA	0.805 (0.432, 1.499)	0.0%
VEN vs PLA	**0.609 (0.404, 0.916)**	0.0%
VIL vs PLA	0.345 (0.099, 1.208)	na
VOR vs PLA	1.569 (0.380, 6.475)	na

Forest plot (vs placebo, the numbers are risk ratios with 95% credible interval)

Treatments are ranked according to their SCURA.

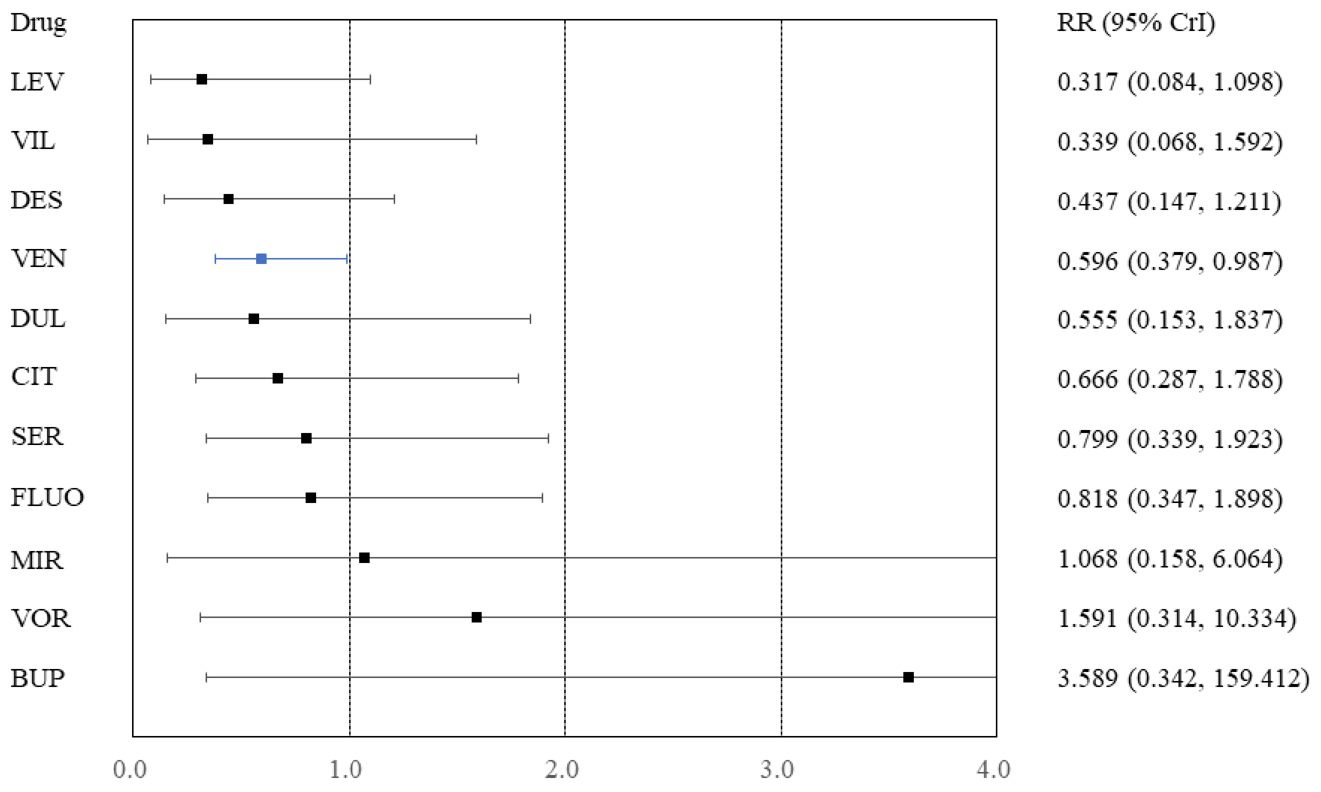

Appendix S6. Headache (K = 23, n = 6934).

League table (risk ratio with 95% credible interval)

PLA																
1.247 (0.481, 3.149)	AGO															
0.792 (0.324, 1.876)	0.631 (0.173, 2.403)	BUP														
1.153 (0.642, 2.104)	0.924 (0.301, 2.899)	1.456 (0.522, 4.350)	CIT													
1.207 (0.745, 2.052)	0.969 (0.335, 2.939)	1.537 (0.565, 4.426)	1.051 (0.485, 2.306)	DES												
1.175 (0.617, 2.244)	0.935 (0.302, 3.028)	1.492 (0.497, 4.556)	1.018 (0.422, 2.466)	0.972 (0.415, 2.172)	DUL											
1.060 (0.425, 2.922)	0.842 (0.222, 3.442)	1.362 (0.388, 5.010)	0.928 (0.302, 2.934)	0.888 (0.299, 2.626)	0.905 (0.295, 2.972)	ESC										
0.963 (0.554, 1.685)	0.764 (0.262, 2.360)	1.223 (0.442, 3.442)	0.841 (0.370, 1.863)	0.800 (0.365, 1.652)	0.821 (0.348, 1.984)	0.907 (0.287, 2.633)	FLUO									
0.408 (0.118, 1.276)	0.327 (0.069, 1.384)	0.519 (0.112, 2.189)	0.350 (0.091, 1.270)	0.337 (0.089, 1.157)	0.345 (0.086, 1.262)	0.379 (0.079, 1.687)	0.423 (0.114, 1.520)	FLUV								
1.380 (0.776, 2.596)	1.113 (0.382, 3.341)	1.756 (0.624, 5.135)	1.194 (0.524, 2.853)	1.149 (0.527, 2.457)	1.178 (0.495, 2.921)	1.287 (0.414, 4.108)	1.424 (0.650, 3.220)	3.338 (0.955, 13.581)	LEV							
0.791 (0.319, 2.042)	0.638 (0.170, 2.422)	1.005 (0.289, 3.586)	0.689 (0.231, 2.081)	0.658 (0.228, 1.898)	0.671 (0.219, 2.136)	0.739 (0.196, 2.698)	0.823 (0.284, 2.366)	1.907 (0.447, 9.330)	0.574 (0.191, 1.722)	MIR						
1.489 (0.598, 3.858)	1.173 (0.319, 4.576)	1.868 (0.518, 6.991)	1.291 (0.435, 3.947)	1.232 (0.414, 3.618)	1.258 (0.408, 4.058)	1.399 (0.355, 5.214)	1.542 (0.535, 4.630)	3.730 (0.814, 17.295)	1.092 (0.341, 3.323)	1.918 (0.489, 7.030)	NEF					

0.954 (0.205, 4.726)	0.785 (0.121, 4.932)	1.248 (0.214, 7.518)	0.830 (0.159, 4.710)	0.801 (0.160, 4.025)	0.811 (0.151, 4.708)	0.905 (0.145, 6.122)	0.992 (0.199, 5.386)	2.377 (0.351, 18.664)	0.697 (0.131, 3.686)	1.223 (0.209, 7.336)	0.654 (0.108, 3.906)	REB				
0.862 (0.487, 1.511)	0.690 (0.228, 2.070)	1.090 (0.392, 3.235)	0.751 (0.332, 1.680)	0.716 (0.327, 1.485)	0.736 (0.310, 1.715)	0.816 (0.259, 2.376)	0.899 (0.413, 1.952)	2.093 (0.588, 8.130)	0.625 (0.274, 1.431)	1.090 (0.369, 3.166)	0.581 (0.189, 1.698)	0.895 (0.164, 4.538)	SER			
1.160 (0.716, 1.892)	0.924 (0.322, 2.744)	1.468 (0.551, 4.052)	1.014 (0.459, 2.161)	0.968 (0.467, 1.861)	0.985 (0.435, 2.224)	1.092 (0.353, 3.084)	1.204 (0.582, 2.515)	2.838 (0.819, 10.913)	0.841 (0.379, 1.769)	1.466 (0.512, 4.138)	0.777 (0.264, 2.215)	1.211 (0.221, 6.027)	1.345 (0.651, 2.784)	VEN		
1.951 (0.874, 5.137)	1.556 (0.449, 6.098)	2.524 (0.726, 9.298)	1.709 (0.612, 5.219)	1.635 (0.604, 4.637)	1.670 (0.584, 5.344)	1.859 (0.501, 7.005)	2.032 (0.734, 6.110)	**4.873 (1.196, 22.351)**	1.422 (0.499, 4.271)	2.487 (0.703, 9.106)	1.317 (0.378, 5.072)	2.021 (0.325, 12.649)	2.271 (0.831, 6.903)	1.698 (0.653, 4.895)	VIL	
0.934 (0.461, 1.898)	0.753 (0.228, 2.459)	1.175 (0.413, 3.712)	0.803 (0.325, 2.026)	0.777 (0.315, 1.792)	0.791 (0.306, 2.103)	0.880 (0.257, 2.797)	0.972 (0.393, 2.430)	2.310 (0.587, 9.146)	0.673 (0.265, 1.741)	1.179 (0.373, 3.896)	0.632 (0.186, 2.090)	0.977 (0.171, 5.499)	1.079 (0.442, 2.712)	0.807 (0.343, 1.943)	0.478 (0.151, 1.391)	VOR

Evaluation of heterogeneity

Network meta-analysis

Between study variance (τ^2): 0.0256

Heterogeneity assessment: Moderate

Pairwise meta-analysis

	Risk ratio (95% confidence interval)	I^2
AGO vs PLA	1.246 (0.575, 2.703)	na
BUP vs PLA	0.801 (0.395, 1.624)	na
CIT vs PLA	1.133 (0.706, 1.819)	0.0%
DES vs PLA	1.176 (0.816, 1.694)	48.5%
DUL vs PLA	1.219 (0.800, 1.858)	48.2%
ESC vs PLA	1.028 (0.457, 2.312)	na
FLUO vs PLA	0.953 (0.616, 1.474)	30.6%

FLUV vs PLA	0.431 (0.153, 1.217)	na
LEV vs PLA	1.359 (0.843, 2.193)	0.0%
MIR vs PLA	0.810 (0.378, 1.735)	na
NEF vs PLA	1.467 (0.674, 3.192)	na
REB vs PLA	0.972 (0.248, 3.813)	na
SER vs PLA	0.872 (0.576, 1.321)	0.0%
VEN vs PLA	1.152 (0.821, 1.617)	0.0%
VIL vs PLA	1.898 (0.927, 3.883)	na
VOR vs PLA	0.941 (0.561, 1.580)	na

Forest plot (vs placebo, the numbers are risk ratios with 95% credible interval)

Treatments are ranked according to their SCURA.

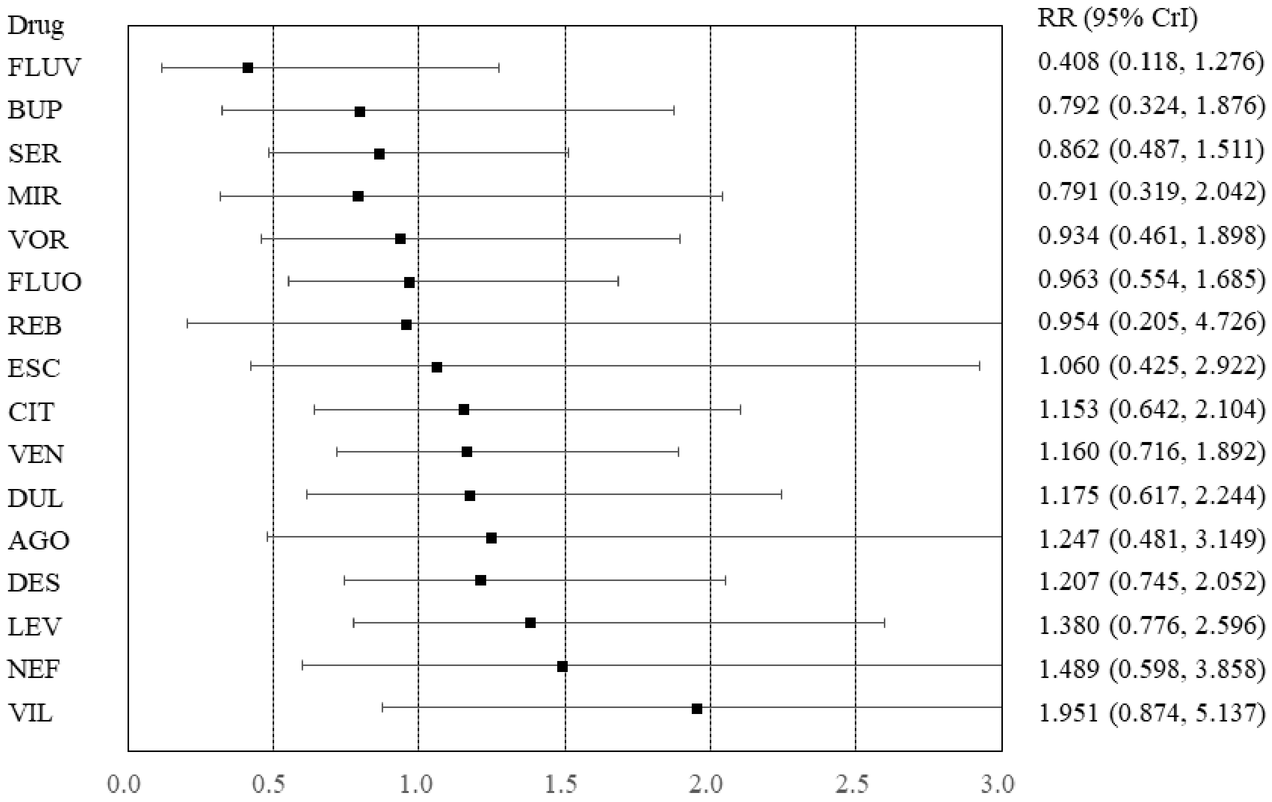

Appendix S7. Somnolence (K = 8, n = 2746).

League table (risk ratio with 95% credible interval)

PLA						
1.128 (0.454, 2.758)	CIT					
1.178 (0.471, 3.149)	1.049 (0.287, 3.933)	FLUO				
0.857 (0.291, 2.530)	0.768 (0.189, 3.027)	0.717 (0.176, 3.018)	MIR			
3.037 (0.368, 59.637)	2.682 (0.276, 58.571)	2.596 (0.247, 55.628)	3.668 (0.340, 72.574)	SER		
0.612 (0.158, 2.226)	0.545 (0.105, 2.638)	0.523 (0.095, 2.492)	0.730 (0.117, 3.955)	0.196 (0.008, 2.759)	VEN	
0.901 (0.195, 5.141)	0.787 (0.134, 5.591)	0.762 (0.124, 5.627)	1.040 (0.159, 7.829)	0.293 (0.011, 4.284)	1.493 (0.192, 12.249)	VIL

Evaluation of heterogeneity

Network meta-analysis

Between study variance (τ^2): 0.073

Heterogeneity assessment: Moderate

Pairwise meta-analysis

	Risk ratio (95% confidence interval)	I^2
CIT vs PLA	1.110 (0.534, 2.308)	0.0%
FLUO vs PLA	1.159 (0.593, 2.265)	na
MIR vs PLA	0.861 (0.378, 1.961)	na
SER vs PLA	2.378 (0.269, 21.010)	na
VEN vs PLA	0.639 (0.215, 1.904)	na
VIL vs PLA	0.863 (0.208, 3.571)	na

Forest plot (vs placebo, the numbers are risk ratios with 95% credible interval)

Treatments are ranked according to their SCURA.

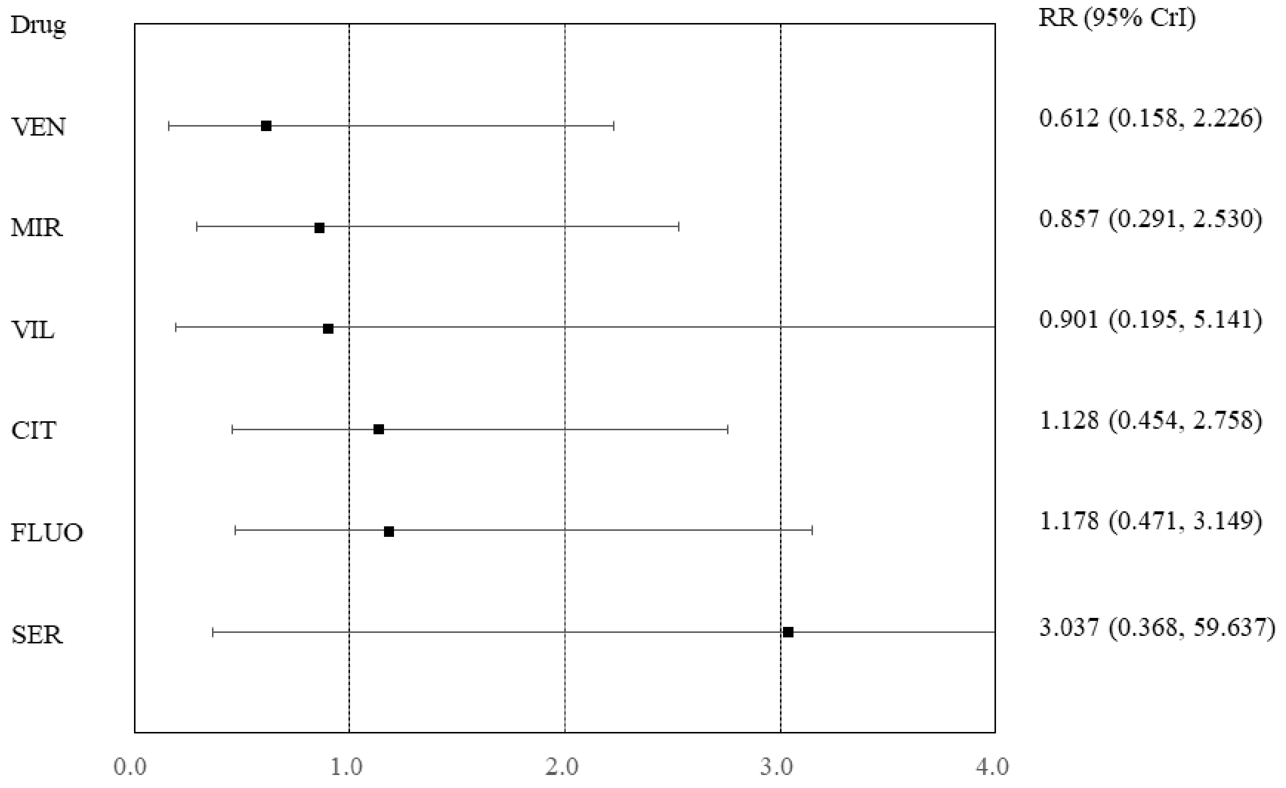

Appendix S8. Insomnia (K = 19, n = 6219).

League table (risk ratio with 95% credible interval)

PLA												
1.006 (0.138, 7.381)	BUP											
1.429 (0.658, 3.970)	1.462 (0.177, 13.550)	CIT										
1.150 (0.345, 3.947)	1.166 (0.112, 11.317)	0.807 (0.168, 3.312)	DES									
0.427 (0.125, 1.405)	0.429 (0.044, 4.185)	0.295 (0.059, 1.159)	0.372 (0.064, 2.010)	DUL								
0.766 (0.198, 2.982)	0.764 (0.072, 7.824)	0.528 (0.096, 2.429)	0.663 (0.103, 4.205)	1.777 (0.299, 11.103)	ESC							
1.539 (0.657, 3.871)	1.539 (0.188, 13.521)	1.081 (0.276, 3.463)	1.353 (0.287, 6.132)	3.617 (0.840, 17.357)	2.020 (0.427, 11.053)	FLUO						
0.980 (0.308, 3.321)	0.956 (0.099, 9.458)	0.663 (0.147, 2.793)	0.839 (0.158, 4.744)	2.310 (0.432, 12.866)	1.277 (0.217, 7.886)	0.627 (0.147, 2.635)	LEV					
1.007 (0.221, 4.266)	0.979 (0.085, 11.143)	0.698 (0.113, 3.615)	0.872 (0.131, 5.787)	2.356 (0.357, 15.589)	1.309 (0.172, 9.102)	0.656 (0.111, 3.624)	1.037 (0.139, 6.516)	REB				
1.233 (0.474, 3.145)	1.238 (0.140, 11.179)	0.852 (0.197, 2.774)	1.086 (0.220, 4.718)	2.860 (0.627, 13.013)	1.627 (0.304, 8.563)	0.790 (0.202, 2.737)	1.272 (0.270, 5.552)	1.218 (0.218, 7.256)	SER			
1.136 (0.335, 3.718)	1.098 (0.110, 10.959)	0.787 (0.154, 3.033)	0.981 (0.172, 5.308)	2.634 (0.478, 13.956)	1.471 (0.233, 9.003)	0.720 (0.160, 3.235)	1.159 (0.201, 6.048)	1.115 (0.164, 7.850)	0.905 (0.201, 4.154)	VEN		
0.568 (0.147, 2.475)	0.566 (0.056, 6.556)	0.391 (0.072, 1.984)	0.496 (0.077, 3.501)	1.333 (0.225, 9.166)	0.753 (0.106, 5.406)	0.366 (0.071, 1.997)	0.590 (0.083, 3.685)	0.569 (0.081, 4.312)	0.463 (0.092, 2.724)	0.515 (0.083, 3.502)	VIL	
3.440 (0.322, 98.847)	3.628 (0.168, 154.636)	2.383 (0.189, 77.457)	3.073 (0.203, 102.355)	8.361 (0.569, 301.829)	4.668 (0.302, 155.393)	2.297 (0.177, 69.567)	3.643 (0.258, 124.941)	3.725 (0.220, 121.160)	2.837 (0.229, 92.028)	3.147 (0.227, 104.014)	6.090 (0.371, 207.374)	VOR

Evaluation of heterogeneity

Network meta-analysis

Between study variance (τ^2): 0.122

Heterogeneity assessment: Moderate

Pairwise meta-analysis

	Risk ratio (95% confidence interval)	I^2
BUP vs PLA	0.986 (0.189, 5.154)	na

CIT vs PLA	1.320 (0.686, 2.541)	39.9%
DES vs PLA	1.151 (0.467, 2.837)	na
DUL vs PLA	0.438 (0.181, 1.056)	na
ESC vs PLA	0.734 (0.259, 2.076)	na
FLUO vs PLA	1.518 (0.781, 2.952)	0.0%
LEV vs PLA	0.877 (0.313, 2.460)	58.6%
REB vs PLA	0.972 (0.293, 3.225)	na
SER vs PLA	1.229 (0.613, 2.464)	0.0%
VEN vs PLA	1.101 (0.471, 2.578)	na
VIL vs PLA	0.591 (0.197, 1.776)	na
VOR vs PLA	2.824 (0.283, 28.185)	na

Forest plot (vs placebo, the numbers are risk ratios with 95% credible interval)

Treatments are ranked according to their SCURA.

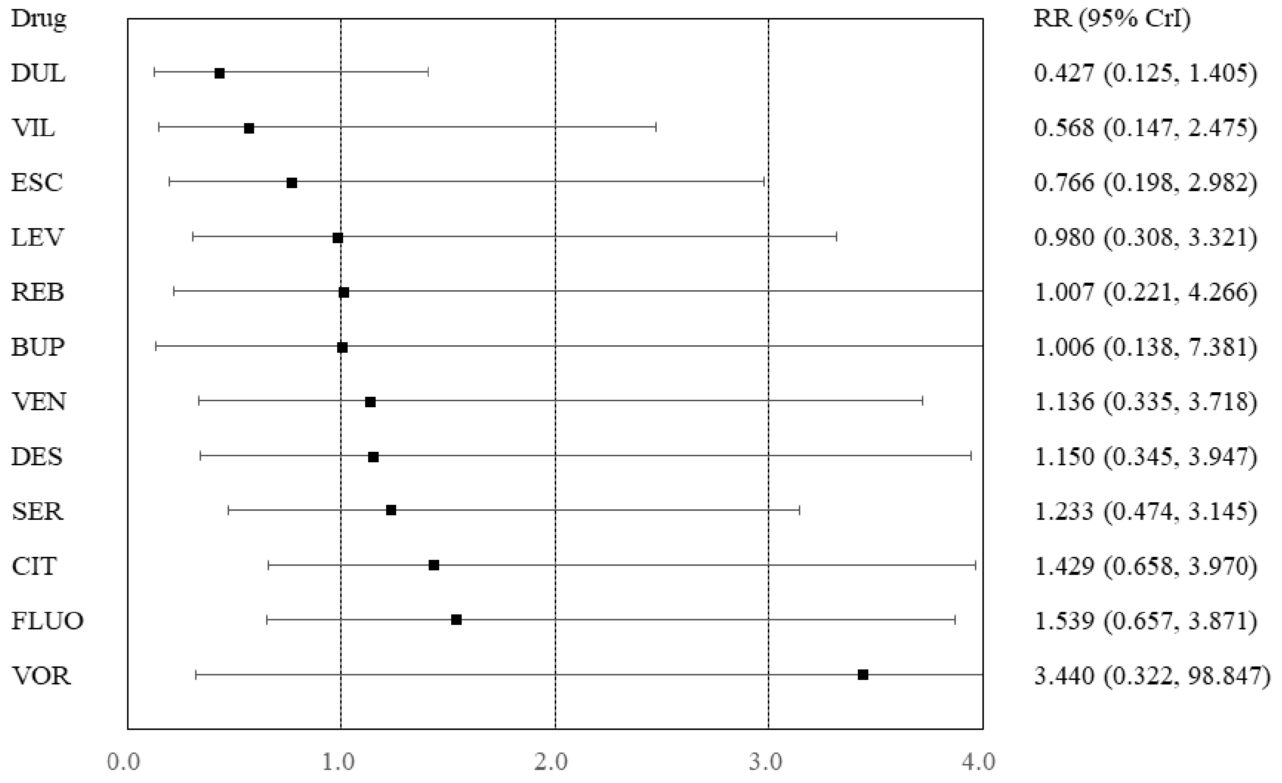

Appendix S9. Dry mouth (K = 12, n = 3913).

League table (risk ratio with 95% credible interval)

PLA						
1.651 (0.518, 6.591)	CIT					
2.184 (0.743, 6.983)	1.317 (0.224, 7.012)	DUL				
1.854 (0.190, 54.937)	1.119 (0.078, 43.020)	0.852 (0.065, 30.310)	LEV			
0.856 (0.261, 2.682)	0.510 (0.087, 2.672)	0.387 (0.070, 1.925)	0.453 (0.012, 5.847)	MIR		
1.438 (0.619, 3.516)	0.860 (0.167, 3.882)	0.657 (0.157, 2.651)	0.774 (0.023, 9.349)	1.705 (0.395, 7.554)	SER	
0.750 (0.241, 2.304)	0.440 (0.079, 2.450)	0.337 (0.070, 1.599)	0.403 (0.010, 4.989)	0.877 (0.184, 4.402)	0.523 (0.124, 2.108)	VEN

Evaluation of heterogeneity

Network meta-analysis

Between study variance (τ^2): 0.104

Heterogeneity assessment: Moderate

Pairwise meta-analysis

	Risk ratio (95% confidence interval)	I^2
CIT vs PLA	1.600 (0.643, 3.984)	na
DUL vs PLA	**2.161 (1.019, 4.585)**	na
LEV vs PLA	1.442 (0.152, 13.708)	na
MIR vs PLA	0.861 (0.378, 1.961)	na
SER vs PLA	1.420 (0.738, 2.734)	0.0%
VEN vs PLA	0.750 (0.358, 1.572)	na

Forest plot (vs placebo, the numbers are risk ratios with 95% credible interval)

Treatments are ranked according to their SCURA.

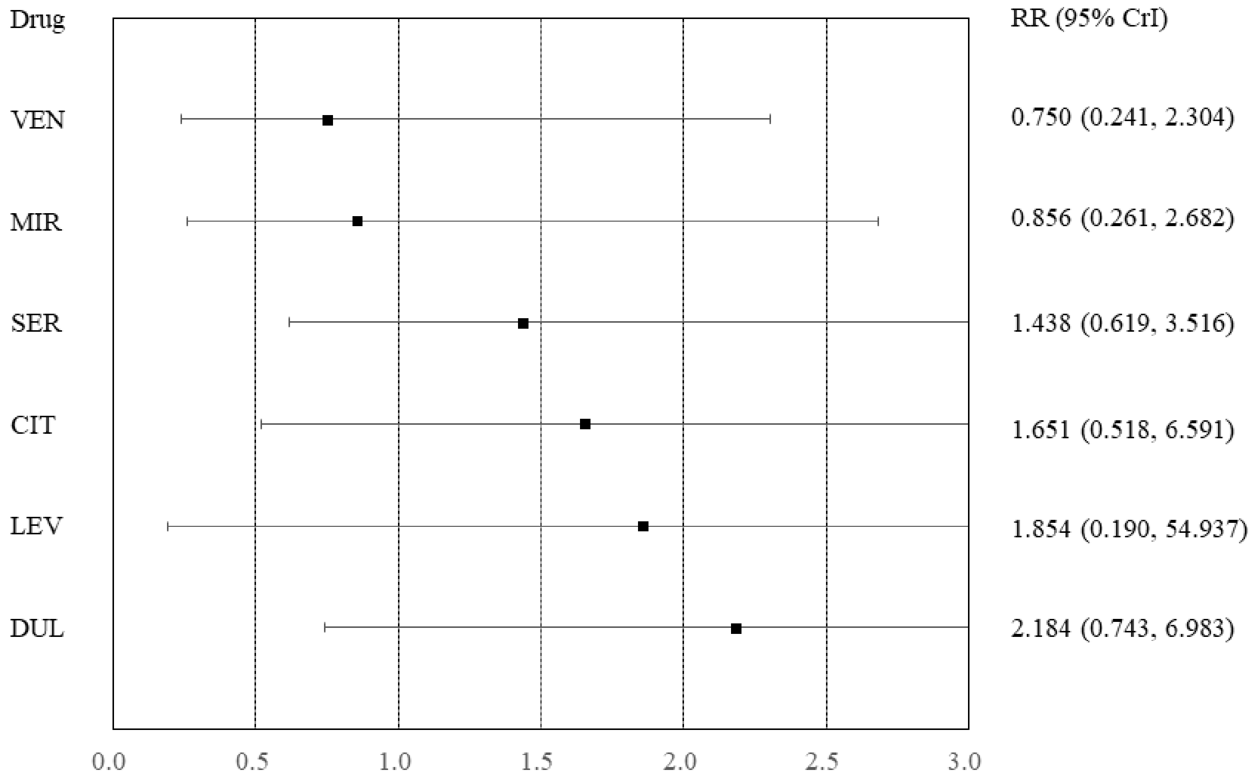

Appendix S10. Constipation (K = 12, n = 3806).

League table (risk ratio with 95% credible interval)

PLA							
0.995 (0.018, 62.910)	BUP						
3.059 (0.458, 22.620)	3.163 (0.032, 277.492)	CIT					
0.716 (0.104, 4.836)	0.727 (0.008, 57.017)	0.229 (0.014, 3.425)	DUL				
0.393 (0.077, 1.978)	0.395 (0.004, 28.523)	0.130 (0.010, 1.726)	0.544 (0.046, 6.936)	LEV			
1.484 (0.224, 10.708)	1.503 (0.015, 114.551)	0.488 (0.030, 7.537)	2.054 (0.140, 34.082)	3.786 (0.314, 47.691)	REB		
0.258 (0.007, 5.199)	0.243 (0.001, 35.970)	0.082 (0.001, 3.127)	0.350 (0.006, 14.257)	0.636 (0.012, 21.469)	0.171 (0.003, 6.213)	SER	
2.086 (0.532, 8.683)	2.058 (0.025, 146.948)	0.681 (0.058, 7.284)	2.883 (0.281, 32.902)	5.318 (0.589, 44.948)	1.406 (0.125, 15.395)	8.149 (0.286, 375.747)	VEN

Evaluation of heterogeneity

Network meta-analysis

Between study variance (τ^2): 0.390

Heterogeneity assessment: High

Pairwise meta-analysis

	Risk ratio (95% confidence interval)	I^2
BUP vs PLA	0.986 (0.062, 15.660)	na
CIT vs PLA	**3.147 (1.201, 8.247)**	0.0%
DUL vs PLA	0.729 (0.260, 2.049)	na
LEV vs PLA	0.439 (0.141, 1.365)	48.4%
REB vs PLA	1.459 (0.533, 3.991)	na
SER vs PLA	0.297 (0.027, 3.241)	na
VEN vs PLA	1.966 (0.930, 4.158)	0.0%

Forest plot (vs placebo, the numbers are risk ratios with 95% credible interval)

Treatments are ranked according to their SCURA.

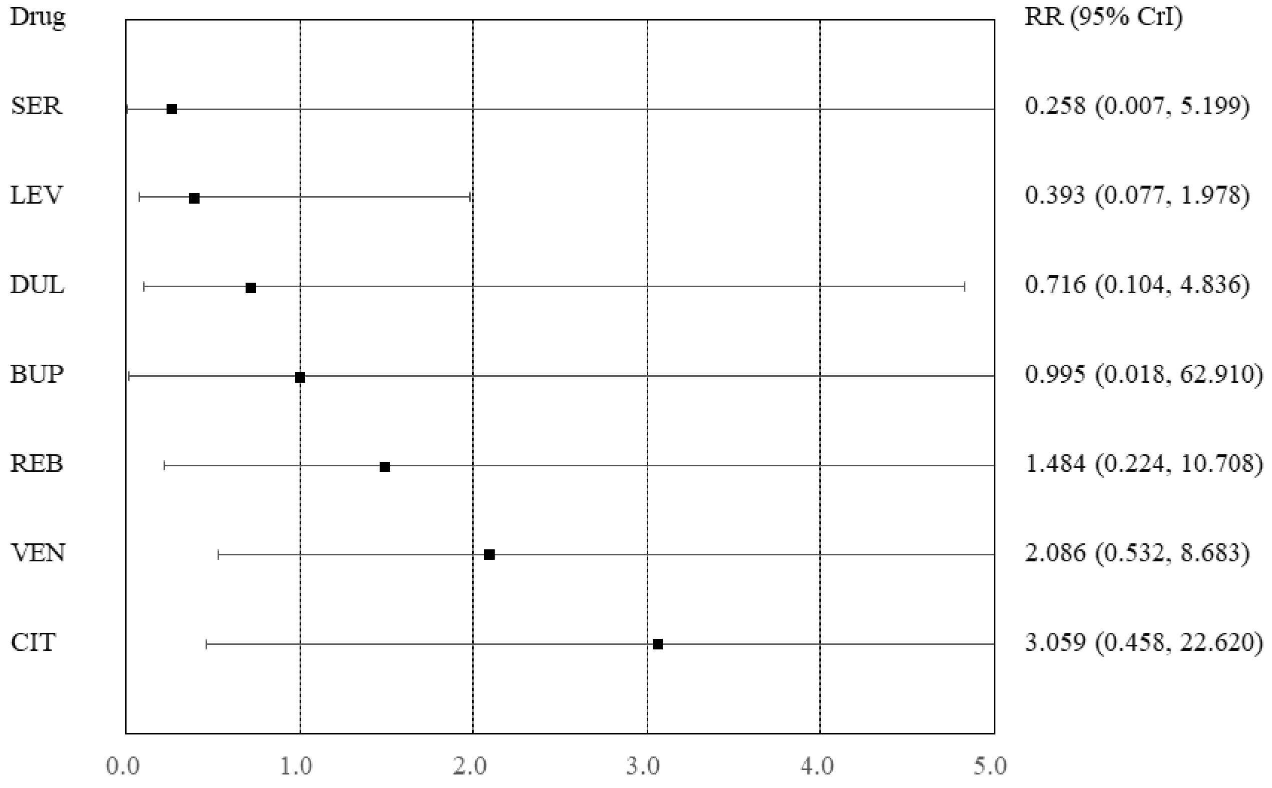

Appendix S11. Sweating (K = 7, n = 1831).

League table (risk ratio with 95% credible interval)

PLA				
0.524 (0.159, 1.570)	CIT			
1.088 (0.204, 6.769)	2.106 (0.287, 17.964)	LEV		
2.289 (0.517, 10.659)	4.398 (0.702, 30.351)	2.146 (0.188, 19.924)	SER	
1.568 (0.568, 4.210)	2.996 (0.690, 13.734)	1.423 (0.180, 10.028)	0.685 (0.111, 4.103)	VEN

Evaluation of heterogeneity

Network meta-analysis

Between study variance (τ^2): 0.171

Heterogeneity assessment: Moderate

Pairwise meta-analysis

	Risk ratio (95% confidence interval)	I^2
CIT vs PLA	0.557 (0.265, 1.168)	0.0%
LEV vs PLA	1.026 (0.205, 5.136)	0.0%
SER vs PLA	2.182 (0.861, 5.530)	na
VEN vs PLA	1.529 (0.888, 2.634)	0.0%

Forest plot (vs placebo, the numbers are risk ratios with 95% credible interval)

Treatments are ranked according to their SCURA.

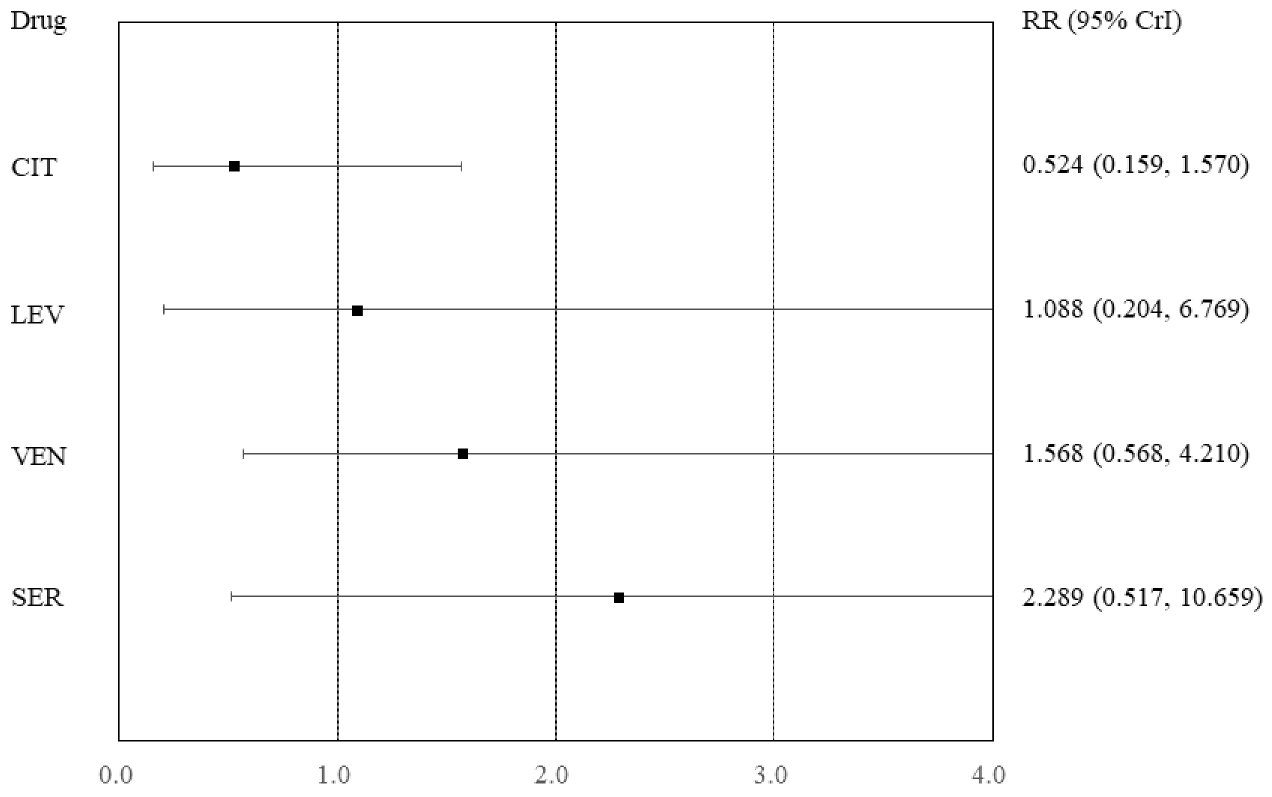

Appendix S12. Weight gain (K = 6, n = 2015).

League table (risk ratio with 95% credible interval)

PLA						
2.417 (0.526, 13.022)	DUL					
1.094 (0.393, 2.928)	0.453 (0.062, 2.668)	MIR				
2.215 (0.656, 8.762)	0.922 (0.110, 7.048)	2.029 (0.429, 10.525)	SER			
1.802 (0.514, 6.391)	0.737 (0.089, 5.401)	1.633 (0.328, 8.398)	0.806 (0.132, 4.788)	VEN		
2.312 (0.628, 10.960)	0.955 (0.111, 8.980)	2.149 (0.413, 13.347)	1.061 (0.150, 7.587)	1.304 (0.206, 9.580)	VIL	
2.509 (0.652, 14.244)	1.033 (0.119, 10.507)	2.308 (0.420, 16.401)	1.138 (0.169, 8.878)	1.431 (0.222, 11.366)	1.081 (0.137, 8.798)	VOR

Evaluation of heterogeneity

Network meta-analysis

Between study variance (τ^2): 0.137

Heterogeneity assessment: Moderate

Pairwise meta-analysis

	Risk ratio (95% confidence interval)	I^2
DUL vs PLA	2.269 (0.599, 8.603)	na
MIR vs PLA	1.096 (0.690, 1.744)	na
SER vs PLA	2.182 (0.861, 5.530)	na
VEN vs PLA	1.753 (0.712, 4.315)	na
VIL vs PLA	2.199 (0.751, 6.446)	na
VOR vs PLA	2.346 (0.707, 7.785)	na

Forest plot (vs placebo, the numbers are risk ratios with 95% credible interval)

Treatments are ranked according to their SCURA.

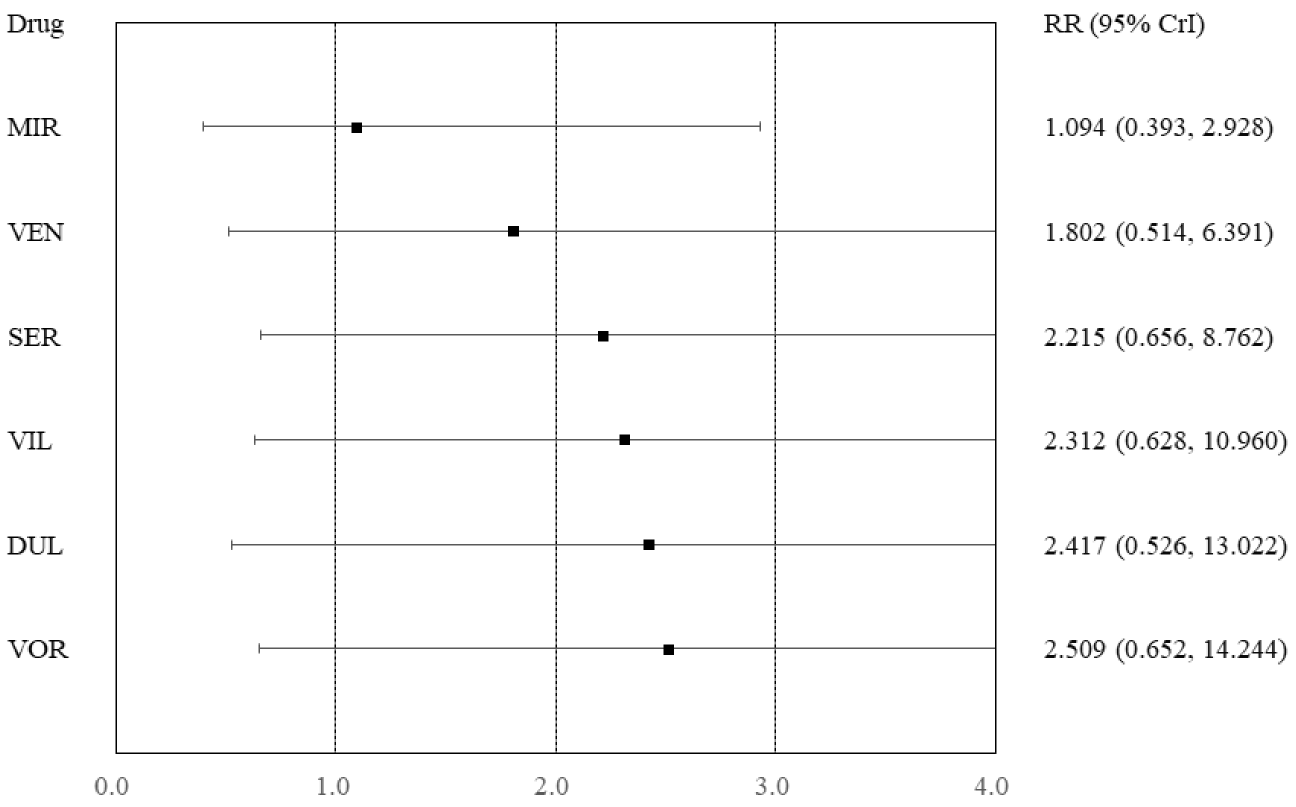

Appendix S13. Sexual dysfunction (K = 6, n = 1519).

League table (risk ratio with 95% credible interval)

PLA					
0.974 (0.078, 12.783)	DUL				
0.916 (0.101, 8.808)	0.926 (0.031, 31.090)	FLUO			
0.779 (0.053, 12.945)	0.806 (0.020, 31.149)	0.860 (0.026, 28.597)	LEV		
8.519 (0.805, 126.908)	8.962 (0.257, 356.349)	9.792 (0.357, 298.325)	11.283 (0.295, 536.570)	SER	
1.606 (0.325, 11.337)	1.676 (0.083, 39.013)	1.786 (0.120, 35.051)	2.077 (0.080, 56.885)	0.187 (0.007, 4.525)	VEN

Evaluation of heterogeneity

Network meta-analysis

Between study variance (τ^2): 0.546

Heterogeneity assessment: High

Pairwise meta-analysis

	Risk ratio (95% confidence interval)	I^2
DUL vs PLA	0.973 (0.200, 4.739)	na
FLUO vs PLA	0.900 (0.390, 2.079)	na
LEV vs PLA	0.721 (0.122, 4.254)	na
SER vs PLA	**7.091 (1.653, 30.417)**	na
VEN vs PLA	1.432 (0.622, 3.299)	0.0%

Forest plot (vs placebo, the numbers are risk ratios with 95% credible interval)

Treatments are ranked according to their SCURA.

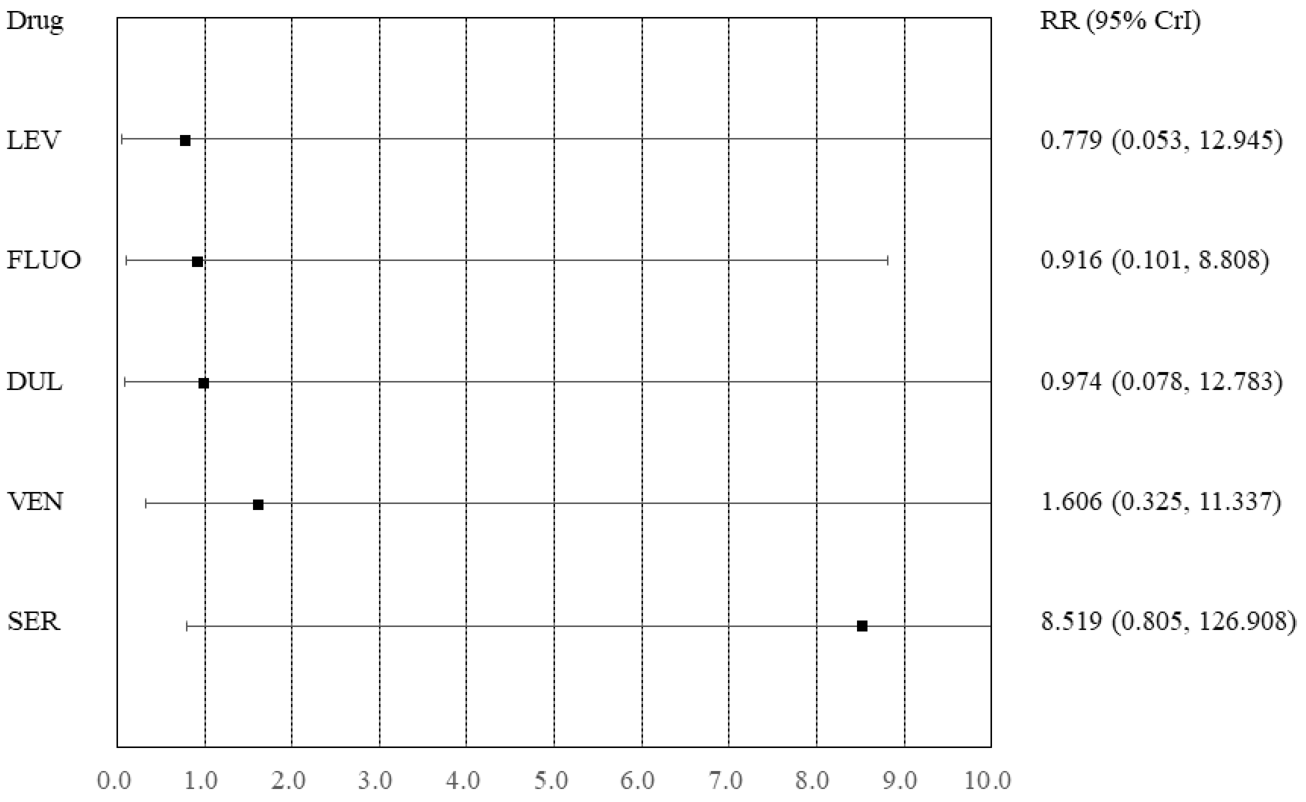

Supplement: Supplementary file 1 — Supplementary materials [file 41380_2022_1824_MOESM1_ESM.pdf]
